# Identification of cardiac-related circulating microRNA profile in human chronic heart failure

**DOI:** 10.18632/oncotarget.6631

**Published:** 2015-12-16

**Authors:** Huaping Li, Jiahui Fan, Zhongwei Yin, Feng Wang, Chen Chen, Dao Wen Wang

**Affiliations:** ^1^ Departments of Internal Medicine and The Institute of Hypertension Tongji Hospital, Tongji Medical College, Huazhong University of Science and Technology, Wuhan, People's Rep. of China

**Keywords:** miRNA profile, heart failure, tissue, circulating, Gerotarget

## Abstract

**Background:**

During chronic heart failure, levels of circulating miRNAs endued with characteristics of diseased cells could be identified as biomarkers. In this study, we sought to identify cardiac-related circulating miRNAs as biomarkers of failing heart.

**Methods:**

Total RNA of plasma and heart samples was extracted from 10 normal controls and 14 patients with chronic heart failure. Microarray was applied for miRNA profiles. Validation and organ/tissue distribution analysis was performed by qRT-PCR. In addition, bioinformatics analysis was performed to understand the critical roles of these cardiac-related circulating miRNAs in heart failure.

**Results:**

Results showed that levels of more than half of the miRNAs dysregulated in heart failed to show any differences in plasma. Meanwhile, more than 90% of the miRNAs dysregulated in plasma remained stable in heart. Four cardiac fibroblast-derived miRNAs (miR-660-3p, miR-665, miR-1285-3p and miR-4491) were found significantly upregulated in heart and plasma during heart failure. These 4 miRNAs strongly discriminated patients from controls, and 3 of them showed significant correlations with LVEF.

**Conclusions:**

This study provides global profiles of miRNAs changes in plasma and failing heart, and using a circulation-tissue miRNA profiling comparison model, we successfully identify 3 cardiac-related circulating miRNAs as potential biomarkers for diagnosis of heart failure.

## INTRODUCTION

Chronic Heart failure (CHF) is described as a complex clinical syndrome resulting from structural or functional disorders of heart. It is a life-threatening disorder worldwide [1]. The recent improvements in cardiac revascularization therapy reduced sudden death due to acute myocardial infarction (AMI) but the number of individuals developing CHF increased steadily [2]. Despite advances in pharmacologic therapy and technologic devices, approximately 50% of CHF patients will die within 5 years [3]. Nowadays, the challenges for CHF including finding highly sensitive, specific, reliable and standardized biomarkers for diagnosis, treatment and prognosis [4].

MicroRNAs (miRNAs) are a class of endogenous, small, non-coding, post-transcriptional RNAs, which play important roles in many essential biological processes [5]. Due to their extraordinary variability of expression patterns and functions across tissues and physiological/pathophysiological states, miRNAs can be powerful diagnostic and therapeutic tools in various disorders, including cardiovascular diseases [6]. The stable presence of miRNAs in circulation, make them promising biomarkers of diseases [7]. A series of studies have demonstrated that cardiomyocytes specific miRNAs (miR-1, miR-208, miR-499, miR-133, etc.) were released into plasma during AMI and correlated with infarct sizes, which may serve as ideal biomarkers [8-11]. However, studies of circulating miRNAs in CHF obtained contradicting results [12-14], probably because of the different detecting approaches used (real-time PCR, microarray, and/or high-throughput sequencing). And the variability of patients' clinical conditions, such as sex, genetic background and medication, may lead to different results. Moreover, the limited number of detected probes and sample size of individual study may induce incomplete profiles. In addition, since CHF is a complex pathophysiologic syndrome which involves multiple organ systems [15, 16], these aberrantly expressed circulating miRNAs in CHF may probably derived from non-cardiac tissues or even blood cells, thereby limiting their clinical practice [17].

In this study, by comparing miRNA expression patterns in heart and circulation, we sought to identify some cardiac-related circulating miRNAs, which could be ideal biomarkers for heart failure.

## RESULTS

### CHF miRNA profiles in plasma and heart

**Figure 1:**
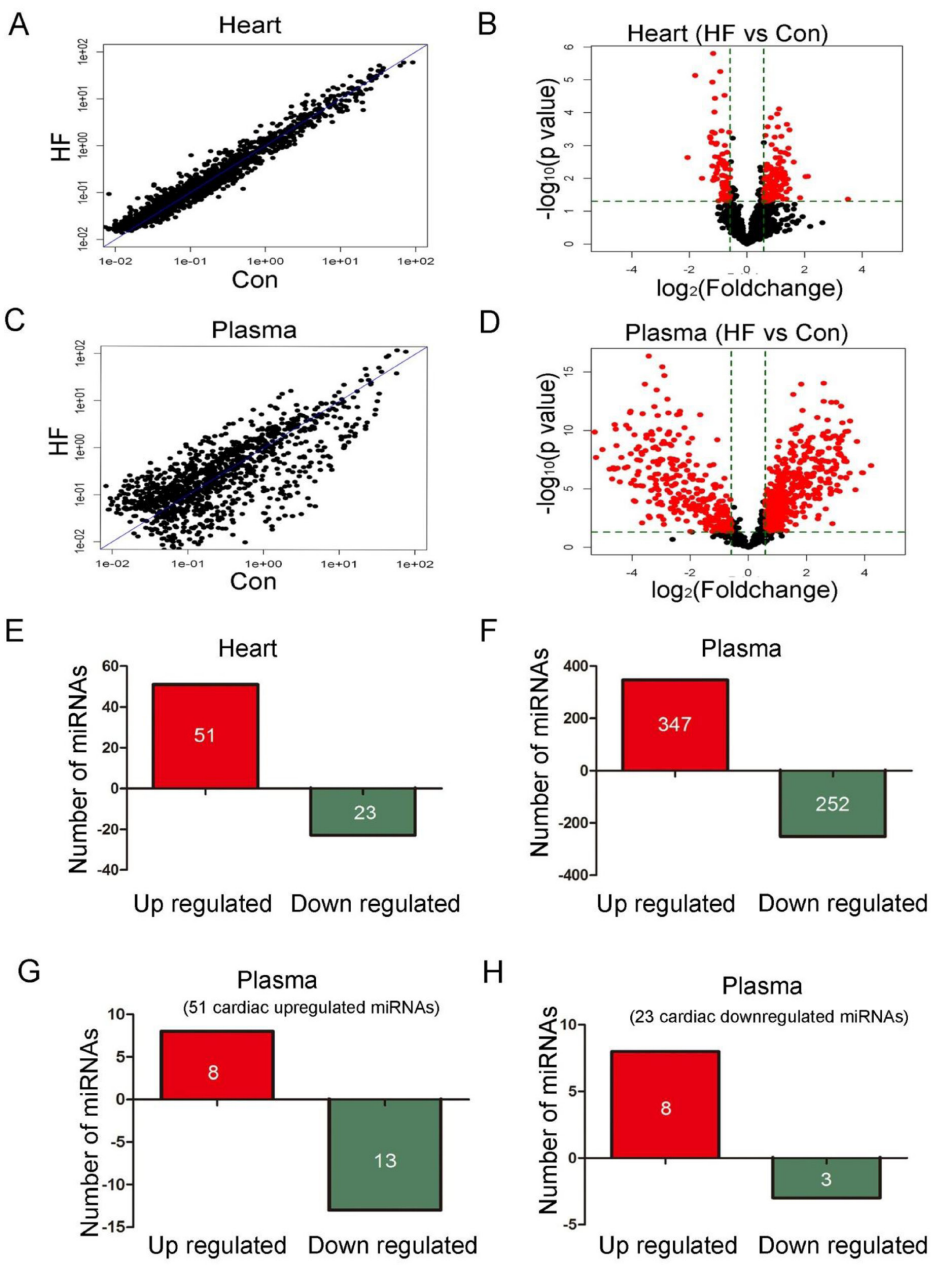
MiRNA profiles in plasma and heart (10 control and 14 CHF patients) **A.** Scatter plot of heart miRNA profiles. **B.** Volcano plot of heart miRNA profiles. **C.** Scatter plot of circulating miRNA profiles. **D.** Volcano plot of circulating miRNA profiles. **E.** 51 out of 3100 miRNAs were up-regulated and 23 miRNAs were down-regulated in heart of CHF patients. **F.** 347 miRNAs were up-regulated and 252 were down-regulated in plasma of CHF patients. **G.** Among the 51 cardiac upregulated miRNAs, 8 miRNAs were upregulated and 13 miRNAs were down-regulated in plasma. **F.** Among the 23 cardiac down-regulated miRNAs, 3 miRNAs were down-regulated and 8 miRNAs were upregulated in plasma.

Heart and plasma samples from 10 control and 14 CHF patients were employed for detecting miRNA profiles. Table S1 provides the baseline characteristics of the patients. Figure 1A and 1B showed the scatter plot and volcano plot of heart miRNA profiles from control and CHF patients. Figure 1C and 1D showed the plots of plasma miRNA profiles. The threshold value for significance used to define up-regulation or down-regulation of miRNAs was a fold change > 2, with a value of *P* < 0.05. Global miRNA profile analysis revealed that 51 out of 3100 miRNAs were up-regulated and 23 miRNAs were down-regulated in heart of CHF patients (Figure 1E). While in plasma, 347 miRNAs were up-regulated and 252 were down-regulated in CHF patients (Figure 1F). By comparing the differently expressed miRNAs in the heart and plasma, we found that during heart failure, among the 51 cardiac-upregulated miRNAs, 8 miRNAs were upregulated, 13 miRNAs were down-regulated in plasma (Figure 1G). Meanwhile, 3 out of the 23 cardiac down-regulated miRNAs showed a decreasing trend and 8 miRNAs were upregulated in plasma (Figure 1F, Tables S2 and S3). However, more than 90% differently expressed circulating miRNAs failed to show any detectable changes in heart during heart failure, which indicated non-cardiac derivation possibility. All altered miRNAs in heart and plasma were listed in Supplemental Tables 4-7.

### Validation of microRNA expression by qRT-PCR

**Figure 2:**
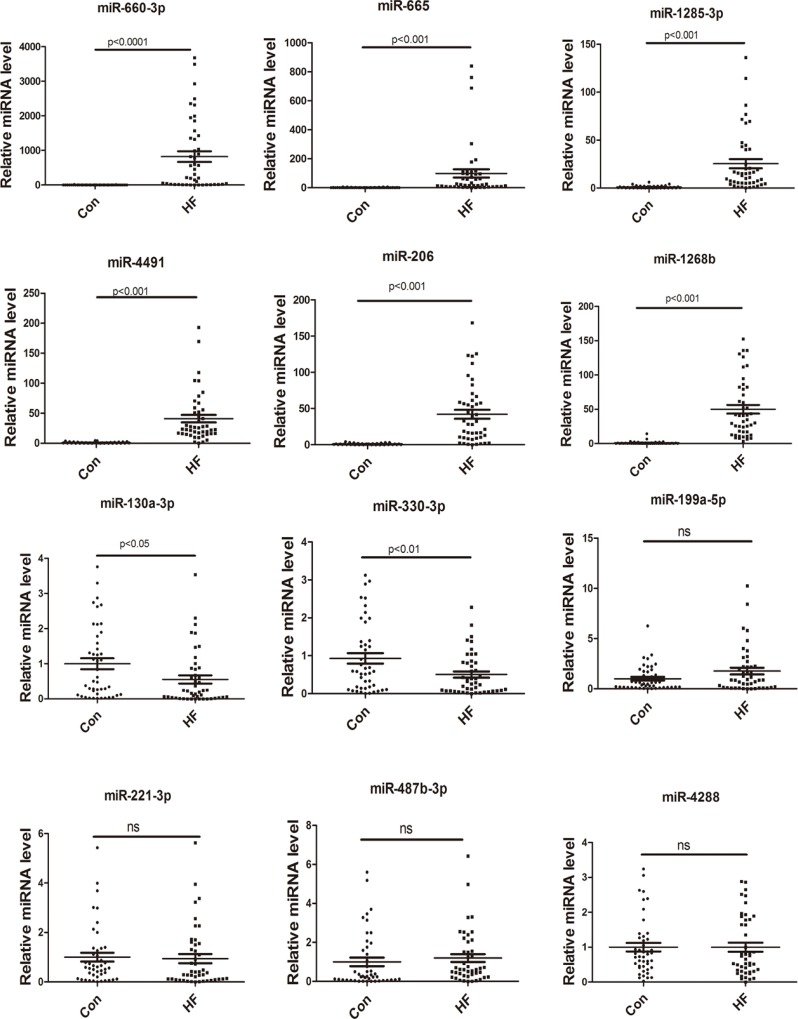
Validation of microRNA expression by real-time PCR Relative expression levels of each miRNA after normalization to U6 (*n* = 45).

Table S8 provided baseline characteristics of the validation cohort. Based on the fold changes, qRT-PCR was performed to validate microarray results on 12 miRNAs, specifically miRNAs up-regulated in both heart and plasma (miR-660-3p, miR-665, miR-1285-3p and miR-4491), down-regulated in heart but up-regulated in plasma (miR-206 and miR-1268b), up-regulated in heart but down-regulated in plasma (miR-130-3p, miR-199a and miR-330-3p), down-regulated in both heart and plasma (miR-221-30, miR-487b-3p and miR-4288), were chosen for validation test in the plasma of 45 control and 45 CHF patients. Figure 2 showed the relative expression levels determined by qRT-PCR of each miRNAs. As a result, 8 of the 12 selected miRNAs (miR-660-3p, miR-665, miR-1285-3p, miR-4491, miR-206, miR-1268b, miR-130-3p and miR-330-3p) were successfully validated in the second cohort.

### Tissue expression patterns of the 8 successfully validated miRNAs

Further, in order to identify cardiac-related miRNAs, we evaluated the tissue distributions of the 8 sucessfully validated miRNAs in normal adult SD rat. Analysis of miRNAs expression revealed that miR-660-3p, miR-665, miR-1285-3p, miR-4491 and miR-130a-3p were relatively cardiac-enriched, while the remaining miRNAs showed a non-cardiac highly expression patterns (Supplemental Figure 1). Given that roughly half of the cells from heart consist of non-myocardial cells, with fibroblasts representing the predominant cell type, then we evaluated the cell distribution of these 8 successfully validated miRNAs. Primary cardiomyocytes and cardiac fibroblasts from neonatal rat hearts were isolated and cultured (Supplemental Figure 2A). Interestingly, all upregulated cardiac-enriched miRNAs (upregulated in circulation) were more abundant in fibroblasts compared to cardiomyocytes, which indicated cardiac fibroblast derivation, while miR-130a-3p, which is also cardiac-enriched but down-regulated in circulation, was more abundant in cardiomyocytes (Supplemental Figure 2B).

### Cardiomyocytes-derived miRNAs in CHF

**Figure 3:**
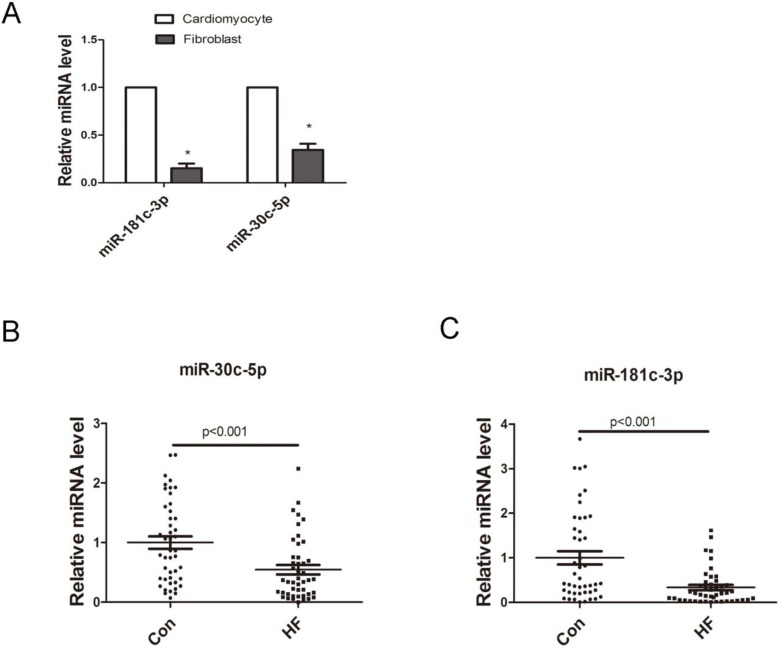
Expression of cardiomyocytes-derived miRNAs **A.** Cell distribution of miR-181c and miR-30c (*n* = 3), *p < 0.05 vs. cardiomyocytes. **B.** Relative expression of miR-181c-3p and miR-30c-5p in plasma of control and CHF patients (*n* = 45).

It was reported that cardiomyocytes-derived miRNAs (miR-1, miR-208, miR-499, miR-133, miR-30c, miR-181, etc.) were released into plasma in acute cardiac damage such as AMI, serving as stable biomarkers [8-11]. Here, we also tried to evaluate their circulating expression levels in CHF. Interestingly, our microarray revealed that during CHF, most reported cardiomyocytes-derived miRNAs were down-regulated in circulation but stayed stable in heart (Table S9). MiR-30c and miR-181c were chosen at random for further investigation. As shown in Figure 3A, miR-30c and miR-181c were more abundant in cardiomyocytes compared to fibroblasts in normal condition. However, results from qRT-PCR suggested that circulating miR-30c and miR-181c level decreased significantly in CHF (Figure 3B and 3C), which is consistent to our profiles data.

### miRNAs as potential biomarkers for CHF

To evaluate whether specific miRNAs might be capable of discriminating CHF patients from controls, we applied ROC analysis for selected 10 successfully validated miRNAs (8 miRNAs were validated in Figure 2 and another 2 miRNAs were validated in Figure 3). The AUC of the 10 selected miRNAs were listed in Table S10. The results showed that 4 cardiac fibroblast derived circulating miRNAs (miR-660-3p, miR-665, miR-1285-3p and miR-4491) all exhibited high accuracy for diagnosis (> 0.9).

### Association between cardiac fibroblast-derived circulating miRNA expression and the severity of CHF

**Figure 4:**
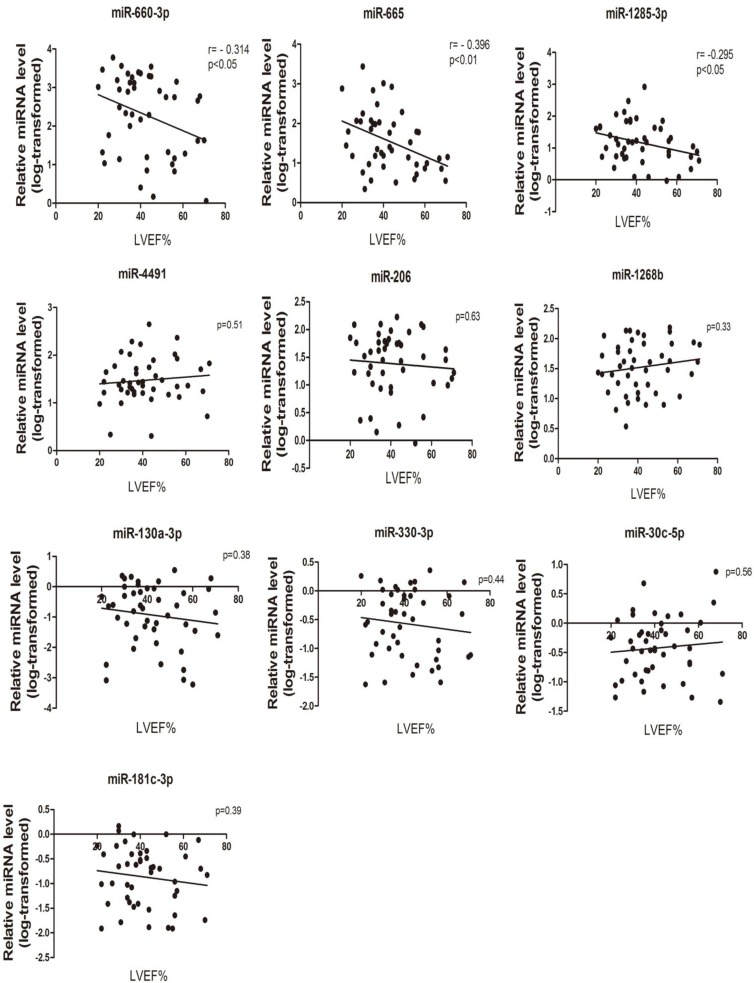
MiRNA expression levels correlation with disease severity The correlation analyses between (log10)-transformed relative miRNA level (CHF patients) and individual measurements of LVEF% (*n* = 45).

Further analysis were made in order to check the possible association between the circulating expression of the 10 successfully validated miRNAs and the clinical parameters from patients, which indicating the severity of CHF. Linear correlation analyses between miRNAs and the LVEF% were performed. Interestingly, as shown in Figure 4, we found significant correlations with LVEF% (*P* < 0.05) for 3 cardiac fibroblast-derived circulating miRNAs, namely miR-660-3p, miR-665 and miR-1285-3p, while the other miRNAs showed no significant correlation.

### GO and pathway analysis of 4 cardiac fibroblast-derived miRNAs

**Figure 5:**
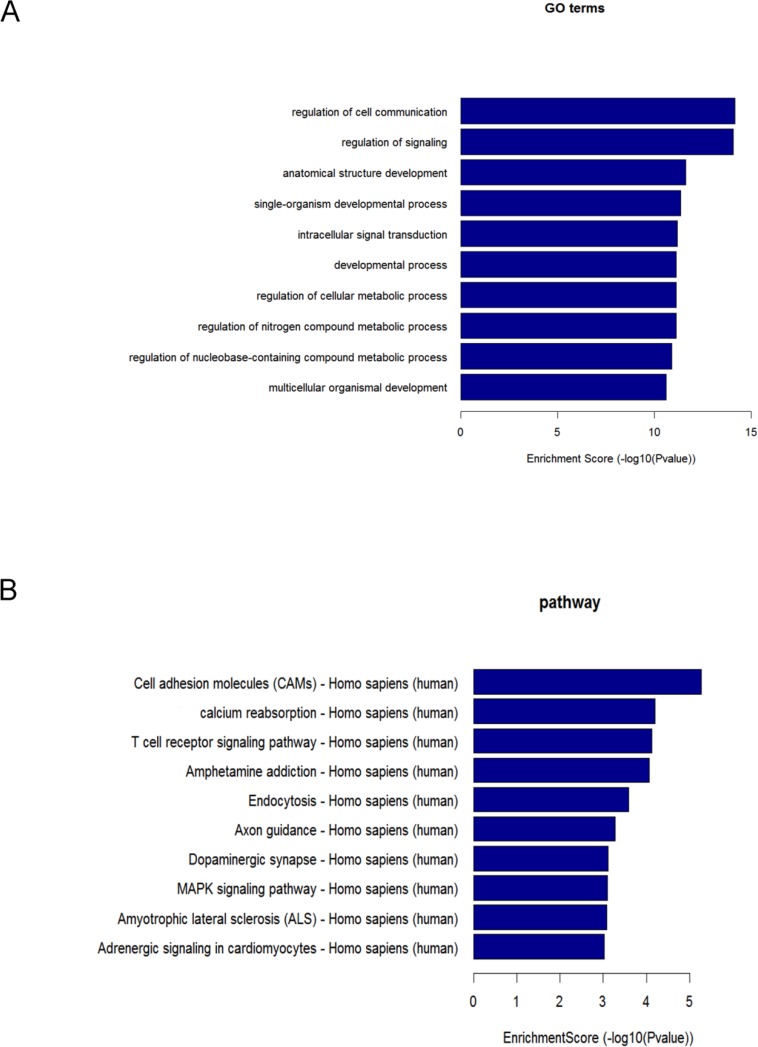
GO and pathway analysis of the 4 cardiac fibroblast-derived miRNAs **A.** GO analysis based on differentially expressed miRNA target genes. The vertical axis is the GO category and the horizontal axis is the negative logarithm of *p* value (−Log p), that represents the significant level of GOs. **B.** Pathway analysis based on differentially expressed miRNA target genes. The vertical axis is the pathway category, and the horizontal axis is the negative logarithm of *p* value (−Log p), that represents the significant level of pathways.

**Figure 6:**
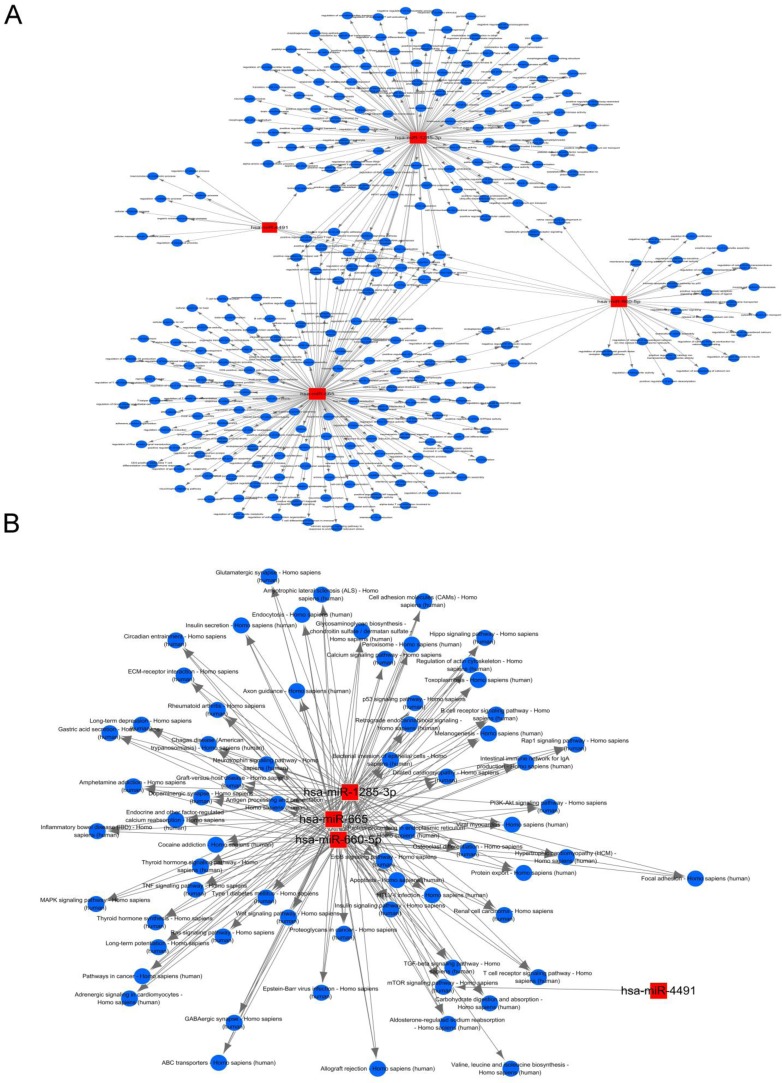
Functional network analysis of the 4 cardiac fibroblast-derived miRNAs **A.** MiRNA-GOs-Network. Red box nodes represent miRNAs, and blue circular nodes represent genes. Edges show the inhibitory effect of microRNAs on GO related genes. **B.** Red box nodes represent miRNAs, and blue circular nodes represent pathways. Edges show the inhibitory effect of microRNAs on pathways.

Among the 10 successfully validated miRNAs, only 4 cardiac fibroblast-derived miRNAs (miR-660-3p, miR-665, miR-1285-3p and miR-4491) were upregulated both in heart and circulation. In order to gain insights into the functions of these cardiac-related circulating miRNAs, target genes of these 4 cardiac fibroblast-derived miRNAs were predicted using TargetScan and miRDB. GO and KEGG pathway analysis were applied to their target pool. As a result, the high enrichment GOs targeted by the CHF-related miRNAs were cell communication, signaling and signal transduction, etc. (Figure 5A). Metabolic processes were also included in the GOs with moderate enrichments. Another functional analysis of miRNAs by KEGG revealed the pathways regulated by these 4 cardiac fibroblast-derived miRNAs (Figure 5B), cell adhesion molecules, calcium reabsorption and T cell signaling pathway were the top 3 pathways. Additionally, miRNA-mRNA gene network analysis integrated these miRNAs and GOs by outlining the interactions of miRNA and GO-related genes (Figure 6A). Meanwhile, miRNA-pathways network analysis revealed all the pathways likely to be regulated by the 4 cardiac fibroblast-derived miRNAs, mainly the 3 LVEF%-associated miRNAs (Figure 6B).

## DISCUSSION

Biomarkers remain an essential tool in daily clinical practice for diagnosis, risk prediction and stratification, and therapeutic targets in diseases. Recent years, several miRNAs have been demonstrated as biomarker in AMI, however, rare circulating miRNAs have been established as suitable biomarkers for CHF [18].

Identifying cardiac-related circulating miRNAs is particularly important in CHF, since levels of circulating miRNAs not always reflect the relative level in heart. However, previous circulating miRNAs profile studies lack of such information. In this study, we demonstrated for the first time that during CHF, more than half of the miRNAs dysregulated in heart failed to display changes in plasma, suggesting rare biomarker potential. Meanwhile, more than 90% of the aberrantly expressed circulating miRNAs showed no expression changes in heart, suggesting non-cardiac tissue derivation.

In our study, 12 miRNAs (not only changed in circulation, but also changed in heart) were selected from microarray profiles for further validation in a second cohort. The results showed that 8 miRNAs were successfully confirmed by qRT-PCR. There are at least two explanations for the discrepancy between the number of differentially expressed miRNAs identified by microarray and qRT-PCR. First, the cohort for microarray was chronic heart failure caused by dilated cardiomyopathy while the cohort for validation included various causes of CHF (valvular, ischemic and dilated cardiomyopathy). Alternatively, some of the microarray probes may have given false-positive signals. Nevertheless, using a combination of microarray and qRT-PCR, we successfully identified 8 miRNAs that were aberrantly expressed during CHF.

Evaluating the tissue distribution of the validated miRNAs, we found that 5 of them (miR-660-3p, miR-665, miR-1285-3p, miR-4491 and miR-130a-3p) were relatively cardiac abundant. Further, we identified the cell distribution of the 8 miRNAs. Interestingly, all 6 upregulated (in circulation) miRNAs were relatively cardiac fibroblast enriched. Interestingly, among these miRNAs, miR-206 was also reported to play important roles via inhibition of TIMP3 in cardiac fibroblasts in heart failure models [19]. Given that CHF is characterized by proliferation and activation of cardiac fibroblasts [20], it can be hypothesized that fibroblasts derived particles (protein and RNA enriched) maybe easier to secret into plasma during CHF, resulting an up-regulation of some fibroblast enriched miRNAs in circulation (Supplemental Figure 3). Further investigation should be applied to illustrate this.

Concerning cardiomyocyte-derived miRNAs, previous studies have revealed that their levels may increase in circulation during AMI [18]. Interestingly, our data suggested that cardiac levels of these miRNAs remain unchanged in CHF patients. Moreover, our results also demonstrated that their circulating levels decreased significantly in CHF, which may indicate impaired secretion of these cardiomyocyte-derived miRNAs into circulation (Supplemental Figure 3). As we know, miRNAs were secreted partly via an exosome dependent way [21, 22], exosome secretion disorder may contribute to the decreased levels of circulating miRNAs. However, cardiac miRNA levels are determined by the combined effects of synthesis, degradation, uptake and secretion. Secretion disorder alone might not be able to influence cardiac miRNA levels, which may explain why these miRNAs were downregulated in circulation yet unchanged in heart (in CHF patients). Though exosome secretion has been demonstrated to be regulated by intracellular calcium and kalium in cultured cortical neurons [23], the mechanisms of exosome secretion in cardiomyocytes/fibroblast have not been clearly understood and further investigation are needed.

In this study, 4 cardiac fibroblast-derived circulating miRNAs (miR-660-3p, miR-665, miR-1285-3p and miR-4491) performed better than other miRNAs in distinguishing CHF and indicating disease severity. It should be noticed that these 4 cardiac fibroblast-derived circulating miRNAs were up-regulated both in heart and plasma suggested by our profiles and qRT-PCR data. The other miRNAs failed to show such characteristics (Table S11). Here, we showed that heart-circulation profile comparison is an efficient way to identify ideal biomarkers in CHF. This method may be extended to the study of other diseases in the future.

In our microarray, some altered miRNAs have been reported before (Supplemental Tables 4-7). The high consistency of reported miRNAs between our data and previous studies indicated reliable data of our microarray. However, most miRNAs provided here have not been well studied before (including miR-660-3p, miR-665, miR-1285-3p and miR-4491), probably due to the following reasons. Firstly, our microarray was performed on the miRCURY LNA Array (version 18.0) station, which contains 3100 capture probes, covering all human miRNAs annotated in miRBase 18.0, as well as all viral miRNAs related to these species. Some of these miRNAs probes were not included in previous studies and microarray versions. Secondly, although these 4 cardiac fibroblast-derived miRNAs are relatively abundant in heart compared to other tissues, their cardiac expression are less than other cardiomyocyte-derived miRNAs (Supplemental Figure 4). Thus, these miRNAs might be detected yet left unstudied due to their limited abundance. However, recent studies demonstrates that miR-21-3p, which has a similar limited abundance to miR-665, miR-1285-3p and miR-4491 (Supplemental Figure 4), plays important role in cardiac hypertrophy, suggesting that miRNA with limited abundance may play important roles in diseases and should not be left ignore [24, 25].

Further bioinformatics analysis aids us in the interpretation of the biological functions of these cardiac-related miRNAs (miR-660-3p, miR-665, miR-1285-3p and miR-4491) in CHF. In this study, KEGG analysis indicated several pathways might be regulated by the 4 cardiac fibroblast-derived miRNAs. Many of these signaling pathways, such as cell adhesion molecules, MAPK pathway, have been demonstrated to participate in heart failure [26, 27]. This represents novel evidences for the modulator roles of miRNAs in CHF. Other pathways, such as calcium reabsorption, endocytosis, neurological signaling pathway, may indicate new mechanisms of CHF, which need to be clarified in the further.

Still, there are following limitations in this study: 1) tissues from early stages of CHF are hardly available and, therefore, studies on human heart samples typically focus on end stage of CHF. Thus our microarray performed on end-stage CHF caused by dilated cardiomyopathy may not include hypertrophy or early stage HF restricted miRNAs; 2) larger studies are needed to confirm the diagnostic capability; 3) gain/lose-of-function and mimics/inhibitor biological studies should be performed to completely validate their functions.

In summary, we compared the expression profiles of circulating and cardiac miRNAs in CHF. And we detected the organ and cell distribution of validated miRNAs. Then, we identified 3 cardiac fibroblast-derived circulating miRNAs (miR-660-3p, miR-665 and miR-1285-3p) significantly upregulated in CHF (in heart and circulation) and correlated to CHF severity, holding promises as diagnostic biomarker for CHF. Moreover, we provided a circulation-tissue profile comparison model, suitable for biomarker identifying for CHF or even other diseases in the future.

## MATERIALS AND METHODS

### Study population

All human hearts and plasma samples were collected from Tongji Hospital (Wuhan, China) between January 2012 and October 2014. The study was approved by the Ethics Review Board of Tongji Hospital and Tongji Medical College. The investigation conforms with the principles outlined in the Declaration of Helsinki (Br Med J 1964; ii: 177). Inform consents were signed by the subjects recruited in the study or by the immediate family members in case of incapacity.

In the screening cohort, left ventricular biopsies and plasma samples were obtained from 14 CHF patients (Table S1) during heart transplantation surgery and 10 control donors (victims of accident), then immediately frozed at −80°C until use. In the validation cohort, plasma samples of 45 CHF patients and 45 controls were recruited.

The inclusion criteria for the CHF group were: chronic heart failure patients, due to idiopathic dilated cardiomyopathy, valvular heart disease or ischemic cardiomyopathy, treated for at least 3 months according to the ACC/AHA guidelines [28], stage C, and clinically stable as judged by the treating HF-specialized cardiologist on the day of recruitment [14]. Exclusion criteria were hypertrophic cardiomyopathy, dilated cardiomyopathy secondary to genetic syndromes, fixed pulmonary hypertension, severe diabetes mellitus with end-organ damage, severe peripheral vascular or cerebrovascular disease, and coexisting active neoplasm. All groups were age- and gender-matched and presented a similar cardiovascular risk profile (Table S8).

### RNA extraction

Total RNA was isolated using TRIzol (Invitrogen) and purified with RNeasy mini kit (QIAGEN) according to manufacturer's instructions. RNA quality and quantity was measured by nanodrop spectrophotometer (ND-1000, Nanodrop Technologies) and RNA Integrity was determined by gel electrophoresis.

### MiRNA microarray

RNA samples were labeled with the Exiqon miRCURY™ Hy3™/Hy5™ power labeling kit (Exiqon, Vedbaek, Denmark) and hybridized on the miRCURY LNA Array (version 18.0) station. Scanning was performed with the Axon GenePix 4000B microarray scanner (Axon Instruments, Foster City, CA). Scanned images were then imported into GenePix Pro 6.0 software (Axon) for grid alignment and data extraction.

### Validation by quantitative real-time PCR (qRT-PCR)

Expression analysis of specific miRNAs was validated by qRT-PCR. Reverse transcription was performed using the SuperScript^®^III First Strand Synthesis Kit (Life Technologies, Carlsbad, CA). Specific miRNA reverse and PCR primers were synthesized by Riobio Co., Ltd (Guangzhou, China). Real-time PCR assays were performed with the SYBR^®^ Select Master Mix (Life Technologies, Carlsbad, CA) on a 7900HT FAST Real-Time PCR System (Life Technologies, Carlsbad, CA). Relative expression levels were calculated with the 2^−ΔΔct^ relative quantification method as previously described [29].

### Isolation of primary cardiomyocytes and cardiac fibroblasts

The investigation conforms to the Guide for the Care and Use of Laboratory Animals published by the US National Institutes of Health (NIH Publication No. 85-23, revised 1985). Hearts were removed from newborn rats (0-3 days), placed in ice-cold Hanks' medium, cut into pieces. The tissues were then incubated in a balanced salt solution containing 0.2% collagenase type 2 (Invitrogen) for 5 minutes at 37°C. The digestion buffer was replaced 6 times, at which point the tissues were completely digested. The collected primary cells were passed through a cell strainer (200 mesh) and then seeded onto petri dishes and incubated for 90min. The supernatant (cardiomyocytes) was collected and plated in DMEM supplemented with 10% FBS, and the adherent cells (cardiac fibroblasts) were cultured under the same conditions as above. Primary cells were confirmed by immunofluorescence staining with antibodies directed against cardiomyocyte-specific marker α2-actinin (ACTN2, Sigma), fibroblast-specific antigen prolyl-4-hydroxylase (P4HB, Acris), endothelial cell marker platelet/endothelial cell adhesion molecule CD31 (PECAM1, Abcam), and smooth muscle α2-actin (ACTA2, Boster, Wuhan, China) as previously described [24].

### MiRNA targets and pathway analysis

Miranda, targetScan and mirbase were utilized for target prediction. The Gene Ontology (GO) Database and the KEGG Pathway Database were used for functional investigation for selected miRNAs.

### Statistical analysis

Demographic and clinical characteristics of the subjects were described as mean±SD. All biomarkers levels were logarithmically transformed for statistical analyses when required. Student's two-sided t-test was used for normally distributed values. For variables without normal distribution, Mann-Whitney U test was performed [30]. The area under the receiver operating characteristic (ROC) curve was calculated to measure the effect strength for candidate miRNAs. Univariable linear and logistics regression analyses were conducted to evaluate the relationships between candidate miRNAs and clinical parameters. *P* < 0.05 (two-tailed) was considered statistically significant.

## SUPPLEMENTARY FIGURES AND TABLES



## Abbreviations

CHF: Chronic Heart failure
AMI: acute myocardial infarction
miRNAs: MicroRNAs
qRT-PCR: quantitative real-time PCR
GO: Gene Ontology
ROC: receiver operating characteristic
