# Supplementary material for: Identification of cardiac-related circulating microRNA profile in human chronic heart failure
Source: Oncotarget. 2015 Dec 16;7(1):33–45. doi: 10.18632/oncotarget.6631 (PMC4807981; doi:10.18632/oncotarget.6631)
Supplement: Supplementary file 1 [file oncotarget-07-0033-s001.pdf]

Identification of cardiac-related circulating microRNA profile in human chronic heart failure

Supplementary Material

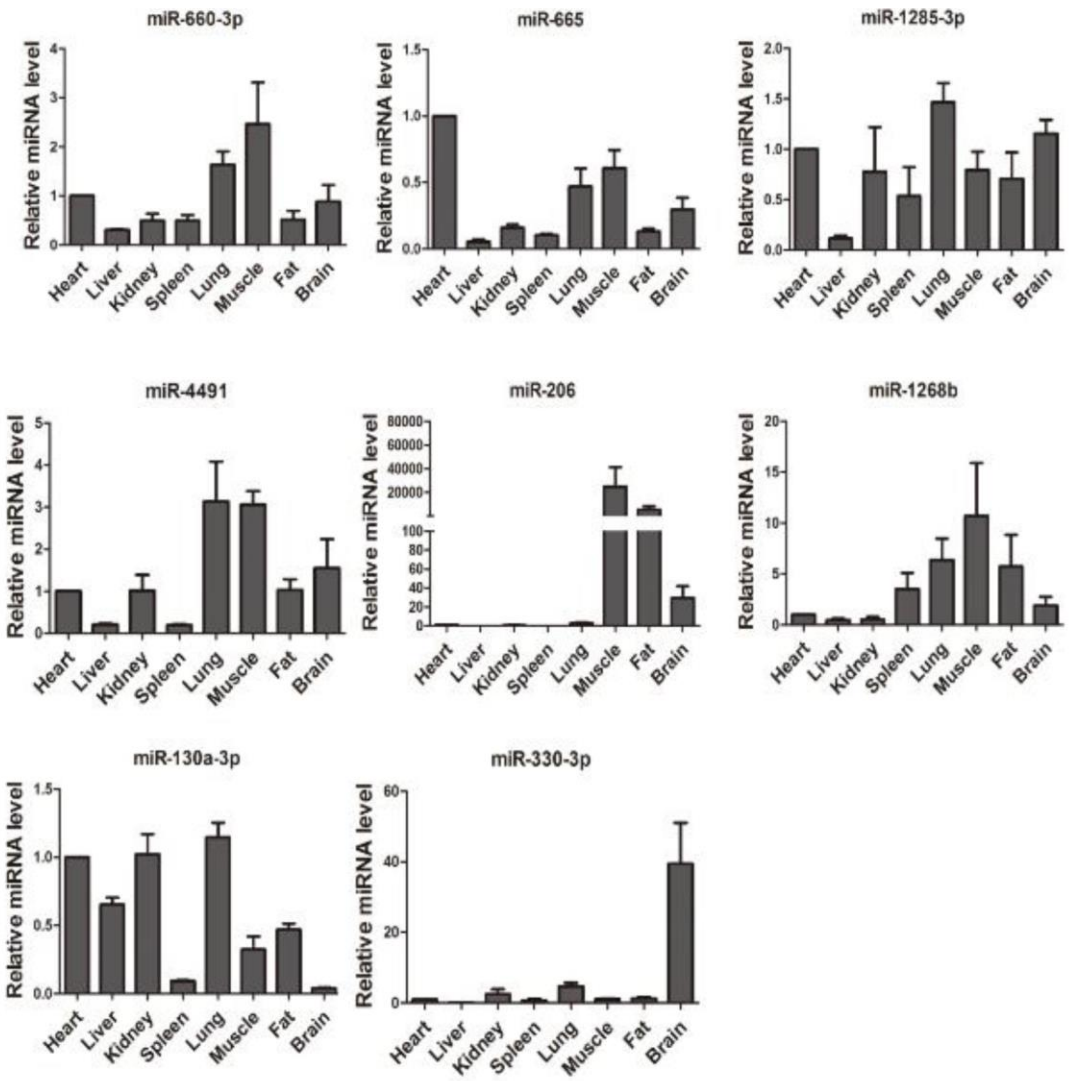

Supplemental Figure 1. Tissue distribution of the 8 successfully validated miRNAs. Relative expression levels of each miRNA after normalization to U6 are shown for miR-660-3p, miR-665, miR-1285-3p, miR-4491, miR-206-3p, miR-1268b, miR-130-3p and miR-330-3p (n=3).

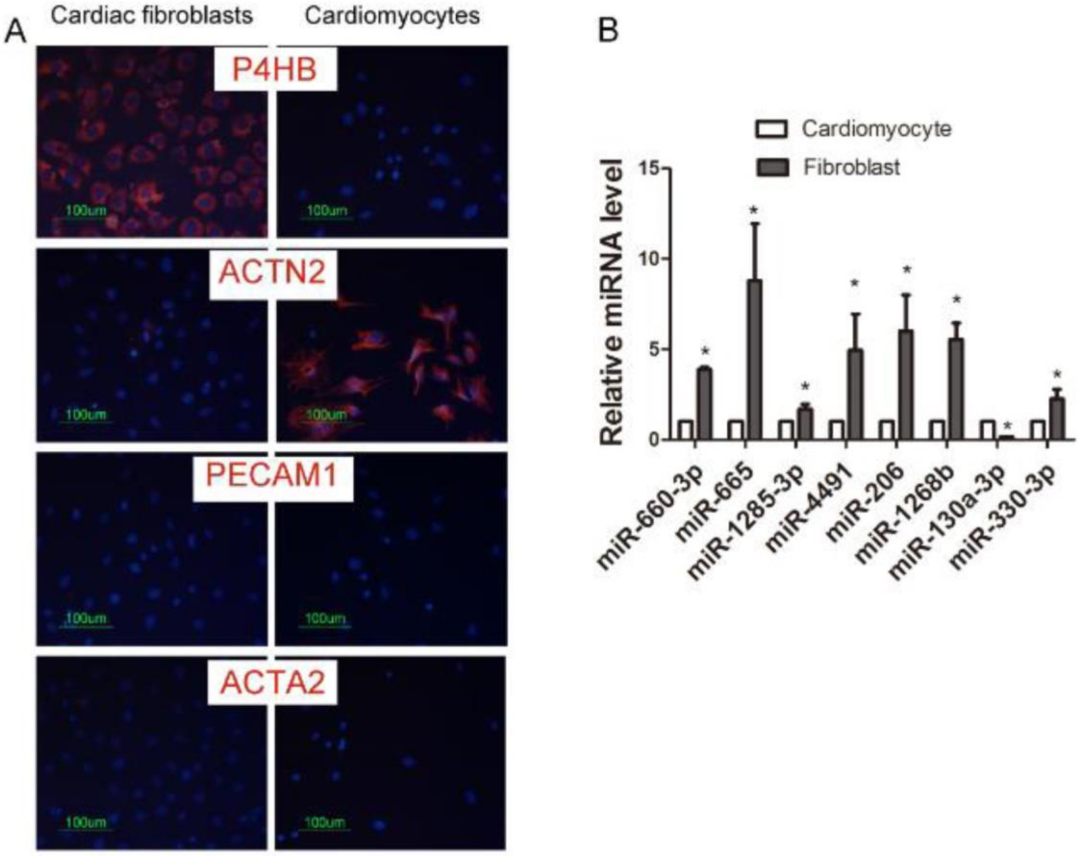

Supplemental Figure 2. Cell distribution of the 8 successfully validated miRNAs. (A) Cells isolated from rat hearts stained with DAPI and antibodies against prolyl4-hydroxylase (P4HB), a2-actinin (ACTN2), CD31 (PECAM1) and smooth muscle a2-actin (ACTA2). (B) Cell distribution of selected miRNAs (n=3), *p<0.05 vs. cardiomyocytes.

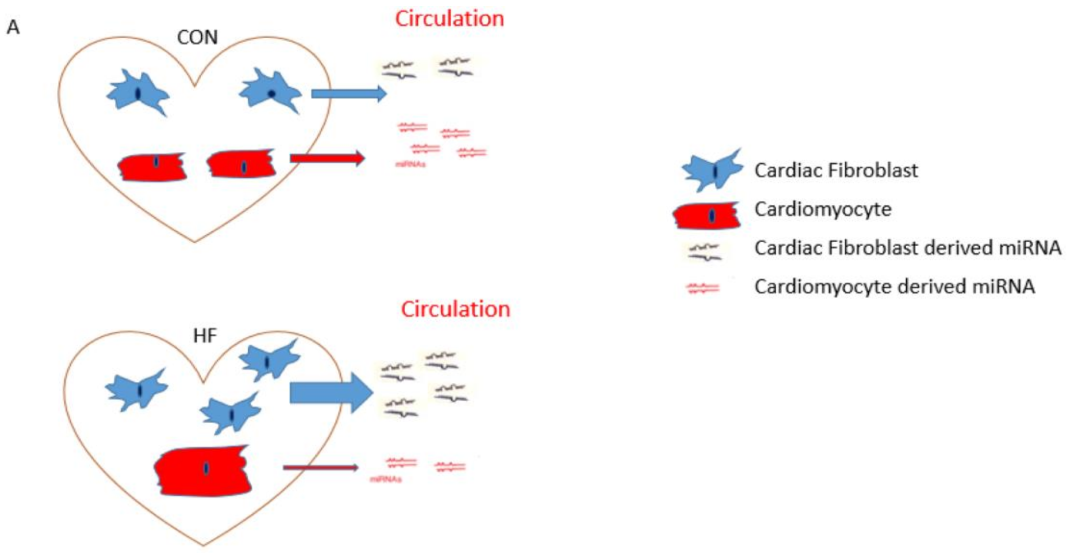

Supplemental Figure 3. Changes of cardiac fibroblast-derived and cardiomyocytes-derived miRNAs secretion pattern in control and chronic heart failure.

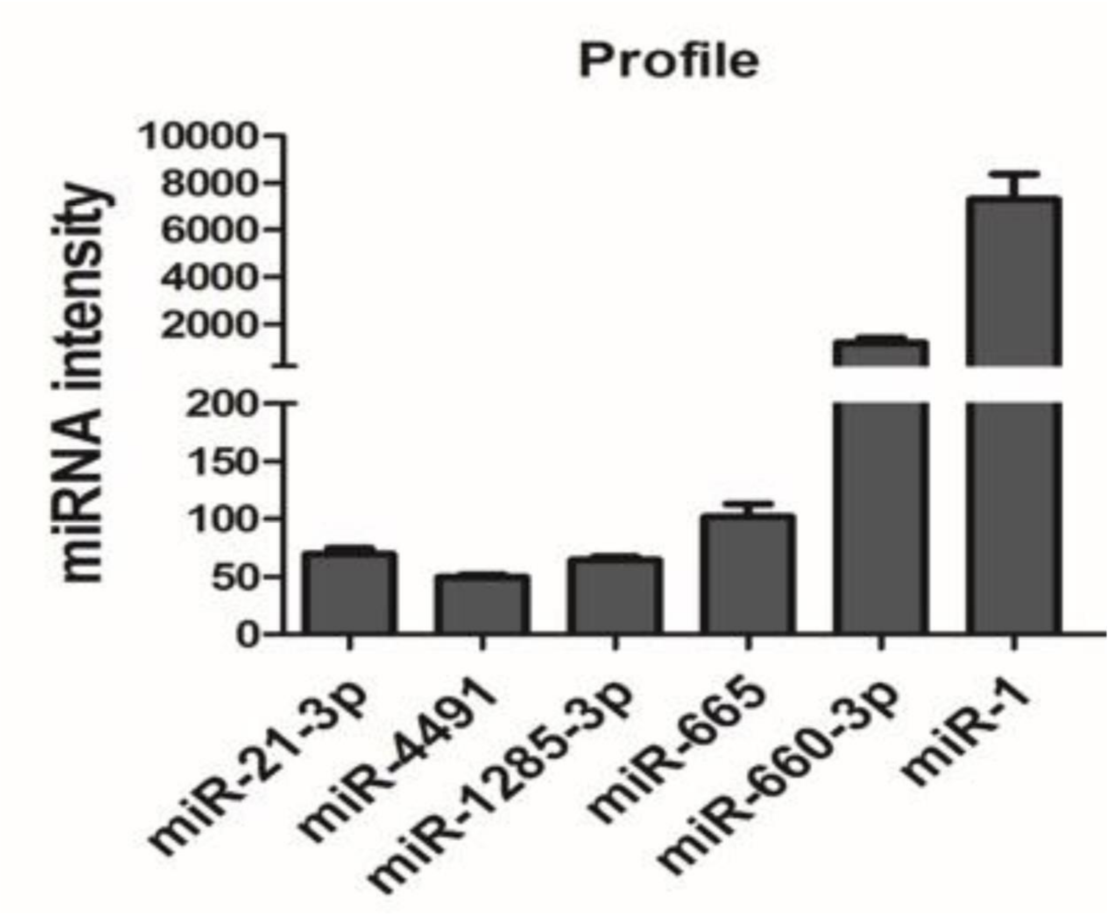

Supplemental Figure 4. Relative abundance of miR-21-3p, miR-4491, miR-1285-3p, miR-660-3p, miR-665 and miR-1 in heart obtained from profiles data.

Supplemental Table 1. Clinical characteristics of patients in the profile cohort.

Patient	Gender	Age(years)	LVEF (%)	LVEDD(mm)	Diagnosis
1	Male	47	17	88	DCM
2	Male	43	20	82	DCM
3	Male	48	24	69	DCM
4	Male	42	9	70	DCM
5	Male	54	23	70	DCM
6	Male	38	18	77	DCM
7	Male	42	28	77	DCM
8	Male	20	40	65	DCM
9	Male	47	23	63	DCM
10	Male	45	41	71	DCM
11	Male	58	31	70	DCM
12	Male	54	19	75	DCM
13	Male	67	43	75	DCM
14	Female	67	24	81	DCM

DCM, dilated cardiomyopathy; LVEF, left ventricular ejection fraction; LVEDD, left ventricular end diastolic diameter

Supplemental Table 2. Profiles data of cardiac-upregulated miRNAs.

miRNA	Fold change in heart (CHF vs Control)	P value	Fold change in plasma (CHF vs Control)	P value
hsa-miR-4491	2.23	0.0226	5.44	2.28E-07
hsa-miR-4503	2.62	0.0002	2.25	0.0003
hsa-miR-1285-3p	2.00	0.0013	5.23	0.0036
hsa-miR-665	4.10	0.0088	6.73	4.41E-08
hsa-miR-4505	2.66	0.0106	2.19	1.23E-05
hsa-miR-1273a	2.24	0.0189	3.82	0.0026
hsa-miR-660-3p	2.48	0.0126	5.36	6.6E-09
hsa-miR-4800-3p	2.22	0.0177	2.92	1.97E-09
hsa-miR-21-3p	3.59	0.0388	0.04	2.01E-07
hsa-miR-130a-3p	2.03	0.0062	0.01	1.45E-07
hsa-miR-A1086	2.07	0.0001	0.35	0.0082
hsa-miR-132-3p	2.23	0.0030	0.22	2.79E-06
hsa-miR-130b-3p	2.27	0.0049	0.29	0.0008
hsa-miR-144-3p	3.07	0.0032	0.23	9.43E-05
hsa-miR-708-3p	2.09	0.0003	0.17	6.52E-10
hsa-miR-451a	2.24	0.0016	0.24	4.18E-08
hsa-miR-199a-5p	2.00	0.0024	0.02	4.21E-08
hsa-miR-21-5p	2.20	0.0023	0.15	2.12E-13
hsa-miR-330-3p	2.57	0.0147	0.07	0.0001
hsa-miR-100-5p	2.40	0.0067	0.49	0.0060
hsa-miR-4436b	2.08	0.038	0.32	0.0076

Supplemental Table 3. Profiles data of cardiac down-regulated miRNAs.

miRNA	Fold change in heart (**CHF vs** Control)	P value	Fold change in plasma (CHF vs Control)	P value
hsa-miR-4288	0.29	7.42E-06	0.32	4.46E-12
hsa-miR-221-3p	0.41	0.0005	0.43	8.23E-08
hsa-miR-487b-3p	0.472	0.0022	0.07	5.35E-06
hsa-miR-1268b	0.24	0.0023	4.17	6.72E-09
hsa-miR-5002-3p	0.43	0.0008	5.70	1.22E-07
hsa-miR-4517	0.44	0.0042	3.00	0.0004
hsa-miR-206	0.48	0.0008	2.56	1.26E-06
hsa-miR-3144-3p	0.46	3.63E-05	3.81	1.65E-08
hsa-miR-1227-3p	048	0.0008	2.16	0.0021
hsa-miR-4664-5p	0.48	0.0045	2.69	0.0068
hsa-miR-3128	0.46	0.0092	2.21	1.35E-05

Supplemental Table 4. Upregulated miRNA in heart of CHF patients (profiles data).

miRNA	Fold change	P value	References
**hsa-miR-21-3p**	3.59	0.038	UP[1]
**hsa-miR-21-5p**	2.19	0.002	ND[2],ND[3],UP[4]
**hsa-miR-100-5p**	2.39	0.006	UP[5],UP[2]
**hsa-miR-124-5p**	2.12	0.003	
**hsa-miR-130a-3p**	2.03	0.006	UP[5],ND[2],UP[4]
**hsa-miR-130b-3p**	2.27	0.004	
**hsa-miR-132-3p**	2.22	0.003	UP[6]
**hsa-miR-142-5p**	2.77	0.0003	
**hsa-miR-144-3p**	3.07	0.003	
**hsa-miR-182-5p**	2.03	0.011	
**hsa-miR-199a-5p**	2.00	0.002	UP[5],UP[2],UP[3]
**hsa-miR-204-5p**	2.66	0.001	UP[5]
**hsa-miR-214-3p**	2.16	7.62E-05	UP[5],UP[2],UP[3]
**hsa-miR-216a-5p**	4.32	0.008	UP[5]
**hsa-miR-217**	11.34	0.042	UP[5]
**hsa-miR-330-3p**	2.57	0.014	UP
**hsa-miR-301b-3p**	2.71	0.001	
**hsa-miR-451a**	2.24	0.001	ND[2]
**hsa-miR-518**	2.35	0.015	
**hsa-miR-660-3p**	2.47	0.012	
**hsa-miR-663a**	2.11	0.031	
**hsa-miR-665**	4.09	0.008	
**hsa-miR-708-3p**	2.09	0.0002	
**hsa-miR-921**	2.49	0.007	
**hsa-miR-1246**	2.49	0.001	
**hsa-miR-1273a**	2.24	0.018	
**hsa-miR-1285-3p**	2.00	0.001	
**hsa-miR-1290**	2.46	0.006	
**hsa-miR-1973**	2.28	0.0005	
**hsa-miR-2114-5p**	2.25	0.021	UP[5]
**hsa-miR-2467-3p**	2.15	0.007	
**hsa-miR-3158-5p**	2.77	0.020	
**hsa-miR-3177-5p**	2.57	0.042	
**hsa-miR-3687**	2.38	0.043	
**hsa-miR-3688-5p**	2.32	0.006	
**hsa-miR-4251**	2.35	0.009	
**hsa-miR-4503**	2.61	0.0002	
**hsa-miR-4436b-3p**	2.07	0.038	

**hsa-miR-4491**	2.22	0.022
**hsa-miR-4508**	2.38	0.003
**hsa-miR-4505**	2.65	0.010
**hsa-miR-4530**	2.51	0.010
**hsa-miR-4688**	2.16	0.008
**hsa-miR-4800-3p**	2.22	0.017
**hsa-miR-5190**	2.05	0.009
**hsa-miRPlus-A1086**	2.07	0.0001
**sv40-miR-S1-5p**	2.28	0.034
**hcmv-miR-US33-3p**	2.36	0.010
**kshv-miR-K12-12-3p**	2.02	0.016
**kshv-miR-K12-6-3p**	2.57	0.025
**kshv-miR-K12-1-5p**	2.00	0.005

UP, upregulated; Down, down-regulated; ND, no difference

Supplemental Table 5. Down-regulated miRNA in heart of CHF patients (profiles data).

miRNA	Fold change	P value	References
**hsa-miR-203b-5p**	0.45	0.011	
**hsa-miR-206**	0.48	0.0008	
**hsa-miR-302b-3p**	0.33	0.009	
**hsa-miR-302d-3p**	0.41	0.0005	Down[5]
**hsa-miR-221-3p**	0.41	0.0005	Down[5]
**hsa-miR-367-3p**	0.43	0.0003	
**hsa-miR-487b-3p**	0.46	0.002	
**hsa-miR-657**	0.45	9.54E-05	
**hsa-miR-933**	0.45	0.0004	
**hsa-miR-1227-3p**	0.47	0.006	
**hsa-miR-1268b**	0.23	0.002	
**hsa-miR-3128**	0.45	0.009	
**hsa-miR-3144-3p**	0.46	3.63E-05	
**hsa-miR-3681-3p**	0.47	0.0009	
**hsa-miR-4255**	0.48	0.010	
**hsa-miR-4288**	0.28	7.42E-06	
**hsa-miR-4517**	0.43	0.004	
**hsa-miR-4521**	0.44	0.011	
**hsa-miR-4650-5p**	0.44	1.59E-06	
**hsa-miR-4664-5p**	0.48	0.004	
**hsa-miR-4699-5p**	0.43	1.18E-05	
**hsa-miR-5002-3p**	0.42	0.0008	
**hsa-miR-5571-5p**	0.49	0.005	

UP, upregulated; Down, down-regulated; ND, no difference

Supplemental Table 6. Upregulated circulating miRNAs in CHF patients (profiles data).

miRNA	Fold change	P value	Reference
**ebv-miR-BART10-5p**	2.487407	0.038518	
**ebv-miR-BART12**	10.10544	1.28E-10	
**ebv-miR-BART15**	2.906815	0.000168	
**ebv-miR-BART16**	8.68136	5.65E-10	
**ebv-miR-BART17-3p**	2.154911	2.44E-05	
**ebv-miR-BART19-3p**	2.141715	2.31E-06	
**ebv-miR-BART19-5p**	2.100169	0.000124	
**ebv-miR-BART2-5p**	2.115982	0.011106	
**ebv-miR-BART4-5p**	8.082383	3.22E-09	
**ebv-miR-BART9-5p**	2.401052	4.83E-07	
**ebv-miR-BHRF1-1**	2.261192	2.49E-05	
**hcmv-miR-UL148D**	2.17782	0.000547	
**hcmv-miR-US25-2-5p**	9.156191	7.14E-10	
**hcmv-miR-US5-2-3p**	5.292159	1.17E-08	
**hsa-let-7a-2-3p**	4.283016	0.000289	
**hsa-miR-1183**	9.094092	8.39E-13	
**hsa-miR-1204**	4.742813	4.23E-07	
**hsa-miR-1207-3p**	2.134171	0.00014	
**hsa-miR-1225-3p**	2.115329	0.00039	
**hsa-miR-1227-3p**	2.158618	0.002092	
**hsa-miR-1229-3p**	2.075263	0.017716	
**hsa-miR-1236-3p**	2.213229	0.000161	
**hsa-miR-1249-3p**	2.043311	1.36E-05	
**hsa-miR-1255b-2-3p**	3.413102	6.01E-08	
**hsa-miR-1257**	5.41877	7.22E-05	
**hsa-miR-1258**	2.72099	2.2E-05	
**hsa-miR-1262**	4.167806	5.83E-05	
**hsa-miR-1264**	3.960086	2.23E-06	
**hsa-miR-1265**	3.744499	2.26E-09	
**hsa-miR-1267**	2.736355	0.000876	
**hsa-miR-1268b**	4.166277	6.72E-09	
**hsa-miR-1272**	2.547148	0.000146	
**hsa-miR-1273a**	3.821383	0.002627	
**hsa-miR-1273d**	2.219281	0.031953	
**hsa-miR-1273e**	2.837645	8.08E-11	
**hsa-miR-1273f**	5.452976	8.87E-06	
**hsa-miR-1273g-3p**	5.215442	1.99E-05	
**hsa-miR-1276**	3.054133	5.3E-06	
**hsa-miR-1284**	3.78234	0.000193	
**hsa-miR-1285-3p**	5.232849	0.003634	

**hsa-miR-1285-5p**	2.455563	1.95E-07	
**hsa-miR-1293**	8.756397	3.39E-09	
**hsa-miR-129-5p**	2.545669	7.68E-05	
**hsa-miR-1296-5p**	3.597907	7.4E-06	UP[7]
**hsa-miR-1299**	8.99203	1.06E-06	
**hsa-miR-1303**	2.442126	0.004385	
**hsa-miR-1304-5p**	2.302671	0.000184	
**hsa-miR-1321**	2.874524	3.98E-08	
**hsa-miR-1322**	4.179342	5.46E-06	
**hsa-miR-135b-5p**	7.423931	0.00957	
**hsa-miR-136-5p**	8.134245	7.63E-08	
**hsa-miR-138-5p**	7.004112	4.85E-10	
**hsa-miR-147a**	8.539801	1.26E-09	
**hsa-miR-147b**	5.989563	1.79E-10	
**hsa-miR-1587**	3.747338	3.64E-08	
**hsa-miR-15a-3p**	2.450658	0.000158	
**hsa-miR-16-1-3p**	2.102115	0.000584	
**hsa-miR-187-3p**	4.424377	4.08E-10	
**hsa-miR-1913**	2.38664	1.63E-05	
**hsa-miR-1915-3p**	2.191916	0.000108	
**hsa-miR-194-3p**	2.544119	1.44E-07	
**hsa-miR-195-3p**	5.76283	2.02E-07	
**hsa-miR-205-5p**	3.157509	0.001045	
**hsa-miR-206**	2.561073	1.26E-06	
**hsa-miR-2113**	6.428722	3.88E-10	
**hsa-miR-216a-3p**	3.083831	4.71E-05	
**hsa-miR-224-3p**	2.464497	0.002957	
**hsa-miR-2681-5p**	2.604816	2.9E-05	
**hsa-miR-296-3p**	2.398336	3.07E-07	
**hsa-miR-296-5p**	2.11056	3.7E-06	
**hsa-miR-297**	10.31108	4.4E-08	
**hsa-miR-29a-5p**	2.802255	4.64E-09	
**hsa-miR-29b-1-5p**	6.032722	9.2E-15	
**hsa-miR-302a-3p**	8.89584	9.69E-09	
**hsa-miR-302e**	2.108823	4.63E-07	
**hsa-miR-3064-5p**	2.838861	8.63E-07	
**hsa-miR-3074-3p**	2.204455	0.000236	
**hsa-miR-3117-3p**	3.062854	1.56E-06	
**hsa-miR-3119**	9.067278	1.35E-08	
**hsa-miR-3120-5p**	7.486032	1.71E-07	
**hsa-miR-3124-3p**	4.652622	8.43E-07	
**hsa-miR-3126-5p**	2.917058	8.28E-14	
**hsa-miR-3127-3p**	2.214521	0.000139	
**hsa-miR-3127-5p**	3.354658	1.87E-07	

**hsa-miR-3128**	2.209125	1.35E-05	
**hsa-miR-3129-5p**	2.862964	3.34E-06	
**hsa-miR-3135a**	3.036654	4.53E-06	
**hsa-miR-3144-3p**	3.809613	1.65E-08	
**hsa-miR-3145-3p**	2.39889	6.93E-08	
**hsa-miR-3146**	2.151491	0.000214	
**hsa-miR-3148**	8.35047	4.02E-09	UP[7]
**hsa-miR-3149**	7.545288	3.11E-10	
**hsa-miR-3150b-5p**	2.127906	0.000258	
**hsa-miR-3152-5p**	3.636848	1.9E-12	
**hsa-miR-3156-3p**	2.246772	1.79E-05	
**hsa-miR-3157-3p**	2.399465	0.001019	
**hsa-miR-31-5p**	2.843622	0.000182	
**hsa-miR-3160-5p**	2.228371	0.000205	
**hsa-miR-3165**	6.400801	5.07E-10	
**hsa-miR-3170**	5.386252	2.95E-05	
**hsa-miR-3171**	4.085072	2.71E-06	
**hsa-miR-3180-5p**	2.21991	1.08E-05	
**hsa-miR-3183**	2.273497	0.001452	
**hsa-miR-3184-3p**	2.578786	4.03E-07	
**hsa-miR-3189-5p**	2.241145	0.00011	
**hsa-miR-3190-5p**	2.413221	3.14E-05	
**hsa-miR-3191-5p**	2.079623	8.04E-10	
**hsa-miR-32-3p**	7.236401	3.97E-13	
**hsa-miR-325**	3.046608	1.5E-08	
**hsa-miR-335-3p**	2.792935	0.001652	
**hsa-miR-33b-5p**	15.26714	4.35E-07	
**hsa-miR-342-5p**	5.61447	2.17E-06	
**hsa-miR-34c-5p**	2.412178	3.47E-05	
**hsa-miR-3591-5p**	5.572873	1.39E-06	
**hsa-miR-3605-3p**	2.067481	0.000543	
**hsa-miR-3606-5p**	5.034147	3.84E-08	
**hsa-miR-3611**	2.960705	6.89E-05	
**hsa-miR-361-3p**	2.101557	3.93E-06	
**hsa-miR-3646**	6.723447	4.94E-08	
**hsa-miR-3649**	5.748201	6.48E-09	
**hsa-miR-3652**	2.561659	4.71E-07	
**hsa-miR-3654**	4.154806	5.75E-07	
**hsa-miR-3657**	4.550566	5.53E-07	
**hsa-miR-3663-5p**	7.179916	1.81E-05	
**hsa-miR-3664-5p**	6.601793	1.29E-11	
**hsa-miR-3667-5p**	4.305872	6.38E-09	
**hsa-miR-3675-3p**	2.137459	3.5E-06	
**hsa-miR-3679-3p**	2.188818	2.72E-06	

**hsa-miR-3680-5p**	18.62661	1.02E-07	
**hsa-miR-3681-5p**	9.835462	2.36E-08	
**hsa-miR-3682-5p**	3.462805	8.6E-10	
**hsa-miR-3689a-3p**	3.080277	7.11E-07	
**hsa-miR-3689b-3p/hsa-miR-3689c**	7.035284	9.38E-06	
**hsa-miR-3690**	2.350586	1.53E-06	
**hsa-miR-3714**	2.103574	1.34E-05	
**hsa-miR-371b-3p**	2.341063	1.03E-06	Down[7]
**hsa-miR-372-3p**	2.318902	5.61E-05	
**hsa-miR-377-5p**	2.077858	1.39E-05	
**hsa-miR-383-5p**	7.701186	3.79E-08	
**hsa-miR-3913-3p**	2.154868	1.21E-05	
**hsa-miR-3914**	2.549329	0.000646	
**hsa-miR-3915**	3.047834	7.13E-07	
**hsa-miR-3920**	3.534529	3E-05	
**hsa-miR-3924**	2.555864	1.74E-06	
**hsa-miR-3935**	4.190024	4.49E-05	
**hsa-miR-3936**	3.143427	5.18E-05	
**hsa-miR-3941**	5.480982	5.66E-08	
**hsa-miR-3943**	2.244744	6.12E-05	
**hsa-miR-3976**	2.411227	5.79E-05	
**hsa-miR-4268**	2.969765	0.000362	
**hsa-miR-4273**	2.726419	8.43E-09	
**hsa-miR-4274**	2.01352	1.92E-06	
**hsa-miR-4279**	2.20314	1.64E-05	
**hsa-miR-4286**	2.030748	6.74E-05	
**hsa-miR-4290**	2.039738	2.49E-09	
**hsa-miR-4292**	2.020139	4.71E-05	
**hsa-miR-4297**	8.098404	1.43E-07	
**hsa-miR-4299**	4.555381	2.73E-08	
**hsa-miR-4302**	11.16708	4.49E-07	
**hsa-miR-4305**	3.044612	0.002101	
**hsa-miR-4307**	4.922762	6.79E-05	
**hsa-miR-4308**	2.77071	9.17E-06	
**hsa-miR-4311**	3.512943	3.76E-08	
**hsa-miR-4312**	2.293805	7.82E-07	
**hsa-miR-4313**	2.195039	0.000201	
**hsa-miR-4324**	2.338523	0.00024	
**hsa-miR-4326**	3.485874	4.19E-05	
**hsa-miR-4329**	3.536211	2.01E-06	
**hsa-miR-433-5p**	2.752218	1.17E-09	
**hsa-miR-4420**	10.2729	1.04E-06	
**hsa-miR-4423-5p**	6.756901	1.41E-06	
**hsa-miR-4424**	3.263135	8.22E-06	

**hsa-miR-4426**	2.408361	2.93E-08
**hsa-miR-4440**	2.50148	8.94E-05
**hsa-miR-4445-3p**	5.315386	0.00191
**hsa-miR-4445-5p**	2.208471	8.3E-07
**hsa-miR-4450**	3.531475	1.1E-14
**hsa-miR-4451**	2.944307	3.87E-07
**hsa-miR-4455**	8.130657	3.94E-13
**hsa-miR-4456**	7.05371	3.02E-07
**hsa-miR-4458**	6.006866	3.28E-13
**hsa-miR-4464**	6.32494	0.000521
**hsa-miR-4473**	6.362573	3.08E-06
**hsa-miR-4477a**	2.217022	8.23E-06
**hsa-miR-4481**	3.387674	3.77E-06
**hsa-miR-4483**	8.772788	4.35E-10
**hsa-miR-4491**	5.43865	2.28E-07
**hsa-miR-4502**	14.99135	4.14E-07
**hsa-miR-4503**	2.247208	0.000293
**hsa-miR-4505**	2.196851	1.23E-05
**hsa-miR-4506**	4.676129	3.2E-05
**hsa-miR-4507**	3.085363	1.93E-10
**hsa-miR-4514**	6.167455	4.21E-08
**hsa-miR-4517**	2.99897	0.000376
**hsa-miR-4518**	9.804438	4.24E-08
**hsa-miR-4520-5p**	11.24782	1.59E-11
**hsa-miR-4524b-5p**	4.547269	3.37E-09
**hsa-miR-4531**	2.319444	1.9E-05
**hsa-miR-4533**	2.031597	2.72E-07
**hsa-miR-4540**	2.910608	7.48E-09
**hsa-miR-4639-3p**	2.948342	7.3E-06
**hsa-miR-4640-3p**	2.326336	2.58E-06
**hsa-miR-4642**	2.16248	0.000327
**hsa-miR-4645-5p**	3.807726	1.13E-08
**hsa-miR-4647**	3.643562	1.78E-05
**hsa-miR-4649-3p**	2.182172	7.51E-06
**hsa-miR-4651**	3.00601	1.56E-08
**hsa-miR-4653-3p**	2.017467	1.72E-05
**hsa-miR-4657**	4.324492	4.28E-09
**hsa-miR-4661-5p**	4.820339	1.22E-05
**hsa-miR-4664-3p**	2.050528	0.000495
**hsa-miR-4664-5p**	2.687848	0.00676
**hsa-miR-4665-3p**	2.321364	5.95E-06
**hsa-miR-4667-3p**	2.053732	7.49E-05
**hsa-miR-4676-5p**	4.086226	3.42E-05
**hsa-miR-4681**	4.032607	5.22E-07

**hsa-miR-4682**	4.909074	1.28E-05
**hsa-miR-4685-3p**	4.883401	1.47E-07
**hsa-miR-4694-5p**	8.337049	4.76E-06
**hsa-miR-4700-3p**	2.291714	9.15E-05
**hsa-miR-4701-3p**	6.941344	3.15E-06
**hsa-miR-4704-5p**	6.803024	3.17E-09
**hsa-miR-4707-3p**	2.115066	0.000622
**hsa-miR-4709-3p**	2.2537	0.000109
**hsa-miR-4710**	2.482125	0.000356
**hsa-miR-4711-3p**	2.106978	5.3E-07
**hsa-miR-4716-5p**	2.235316	2.28E-06
**hsa-miR-4717-5p**	2.251157	0.002092
**hsa-miR-4722-3p**	2.023881	0.013173
**hsa-miR-4722-5p**	5.715518	1.21E-07
**hsa-miR-4723-3p**	2.171927	5.29E-05
**hsa-miR-4725-5p**	5.886335	2.73E-10
**hsa-miR-4726-5p**	2.13618	8.39E-06
**hsa-miR-4728-3p**	2.199693	5.03E-06
**hsa-miR-4730**	12.78483	1.2E-05
**hsa-miR-4731-3p**	2.275194	6.74E-06
**hsa-miR-4732-3p**	2.264644	1.76E-06
**hsa-miR-4735-5p**	2.061846	1.11E-06
**hsa-miR-4742-3p**	2.163039	1.12E-05
**hsa-miR-4749-3p**	2.119412	1.55E-05
**hsa-miR-4752**	2.151855	0.012891
**hsa-miR-4756-5p**	2.77305	0.001159
**hsa-miR-4758-3p**	2.044499	0.000328
**hsa-miR-4765**	3.471019	3.94E-11
**hsa-miR-4768-5p**	3.798824	3.2E-06
**hsa-miR-4774-5p**	2.531993	0.001185
**hsa-miR-4780**	2.792608	0.00014
**hsa-miR-4787-3p**	2.021997	0.000151
**hsa-miR-4791**	2.860953	1.24E-05
**hsa-miR-4795-5p**	3.20665	7.34E-11
**hsa-miR-4796-5p**	7.900793	2.93E-10
**hsa-miR-4800-3p**	2.915018	1.97E-09
**hsa-miR-4803**	6.625456	1.22E-10
**hsa-miR-483-3p**	2.925676	6.68E-05
**hsa-miR-490-5p**	2.276884	0.000534
**hsa-miR-495-5p**	2.899106	5.29E-07
**hsa-miR-5002-3p**	5.700708	1.22E-07
**hsa-miR-5002-5p**	3.407288	4.44E-07
**hsa-miR-5006-3p**	3.788596	1.64E-06
**hsa-miR-500a-5p**	4.24694	2.59E-09

**hsa-miR-5010-3p**	3.110975	5.87E-11	
**hsa-miR-501-5p**	4.1136	5.94E-06	
**hsa-miR-502-5p**	10.38414	4.73E-08	
**hsa-miR-504-5p**	4.974674	2.19E-05	
**hsa-miR-5093**	5.764824	3.42E-08	
**hsa-miR-5095**	7.113629	2.87E-05	
**hsa-miR-514b-3p**	10.27154	1.2E-06	
**hsa-miR-5187-3p**	2.901153	8.7E-05	
**hsa-miR-5189-5p**	3.142949	1.23E-06	
**hsa-miR-518b**	2.082221	0.000206	
**hsa-miR-5193**	2.860376	3.72E-08	
**hsa-miR-5194**	4.110586	1.01E-06	
**hsa-miR-5195-5p**	2.165397	0.001282	
**hsa-miR-5196-3p**	2.868302	4.03E-06	
**hsa-miR-548ag**	2.361918	0.000328	
**hsa-miR-548an**	3.986634	2.01E-08	
**hsa-miR-548ao-3p**	3.221756	4.07E-07	
**hsa-miR-548b-5p**	3.035519	2.17E-09	
**hsa-miR-550b-3p**	4.498327	9.38E-07	
**hsa-miR-553**	2.024479	4.57E-05	
**hsa-miR-5572**	4.21134	7.45E-10	
**hsa-miR-5580-5p**	7.66356	5.77E-07	
**hsa-miR-5581-3p**	3.271859	1.08E-06	
**hsa-miR-5582-3p**	13.28573	8.74E-10	
**hsa-miR-5582-5p**	2.133176	7.18E-07	
**hsa-miR-5584-3p**	2.165683	3.57E-05	
**hsa-miR-5585-3p**	3.639805	0.000372	
**hsa-miR-5681b**	2.353665	6.69E-08	
**hsa-miR-5684**	4.321055	3.31E-06	
**hsa-miR-5689**	2.210945	0.002263	
**hsa-miR-5692c**	7.857415	2.01E-07	
**hsa-miR-5699-3p**	2.51949	3.77E-07	
**hsa-miR-5704**	6.459919	3.62E-07	
**hsa-miR-574-5p**	5.170173	7.84E-12	
**hsa-miR-595**	8.765417	1.65E-05	UP[7]
**hsa-miR-610**	10.73957	1.2E-10	
**hsa-miR-611**	2.269008	0.001074	
**hsa-miR-615-3p**	3.220966	9.83E-07	
**hsa-miR-615-5p**	2.253286	0.013358	
**hsa-miR-625-3p**	2.065208	9.78E-07	
**hsa-miR-632**	3.087	0.011861	
**hsa-miR-634**	2.388046	2.76E-08	
**hsa-miR-635**	2.110965	1.76E-05	
**hsa-miR-642b-5p**	2.272194	7.2E-08	

**hsa-miR-645**	5.483309	7.1E-06	
**hsa-miR-647**	9.420956	2.73E-10	
**hsa-miR-653-5p**	3.649289	0.000947	
**hsa-miR-659-5p**	2.146775	4.8E-05	
**hsa-miR-660-3p**	5.358968	6.6E-09	
**hsa-miR-664a-3p**	2.558522	1.39E-06	
**hsa-miR-664b-3p**	2.811798	3.19E-08	
**hsa-miR-665**	6.725905	4.41E-08	
**hsa-miR-670-5p**	3.530755	9.52E-07	
**hsa-miR-718**	2.15613	0.004179	
**hsa-miR-7-2-3p**	2.091925	3.5E-08	
**hsa-miR-760**	2.834944	9.6E-06	
**hsa-miR-761**	4.063908	1.28E-09	
**hsa-miR-764**	7.371863	2.1E-07	
**hsa-miR-767-3p**	2.263647	0.00231	
**hsa-miR-769-3p**	2.408953	0.000266	
**hsa-miR-885-3p**	4.861341	2.56E-08	
**hsa-miR-885-5p**	4.609738	3.72E-10	UP[8]
**hsa-miR-891a-5p**	2.653416	4.26E-07	
**hsa-miR-892a**	9.415241	2.19E-11	
**hsa-miR-920**	8.565672	1.11E-08	
**hsa-miR-9-3p**	2.208036	0.006593	
**hsa-miR-940**	2.139499	1.46E-05	
**hsa-miR-943**	2.094493	2.01E-05	
**hsa-miRPlus-A1087**	5.709624	7.79E-09	
**hsa-miRPlus-C1066**	3.15542	4.33E-05	
**hsa-miRPlus-G1246-3p**	2.239942	0.000304	
**hsv1-miR-H1-3p**	2.072196	0.000304	
**hsv1-miR-H4-3p**	3.055827	3.62E-05	
**hsv1-miR-H6-3p**	2.037027	2.56E-07	
**hsv1-miR-H7-3p**	2.07696	4.12E-05	
**hsv1-miR-H8-3p**	2.1947	1.14E-06	
**hsv2-miR-H20**	2.1626	0.007325	
**hsv2-miR-H24**	2.053781	6.24E-06	
**hsv2-miR-H9-3p**	2.161794	0.000467	
**kshv-miR-K12-10a-3p**	2.33874	9.69E-07	
**kshv-miR-K12-10b**	2.284871	9.79E-07	
**kshv-miR-K12-1-3p**	8.208424	1.29E-05	
**kshv-miR-K12-1-5p**	8.430126	4.23E-09	
**kshv-miR-K12-5-5p**	5.086253	1.5E-07	
**kshv-miR-K12-8-5p**	2.240374	2.97E-05	
**kshv-miR-K12-9-5p**	2.087056	0.000151	
**sv40-miR-S1-3p**	2.861499	0.001577	

UP, upregulated; Down, down-regulated; ND, no difference

Supplemental Table 7. Down-regulated circulating miRNAs in CHF patients (profiles data).

miRNA	Fold change	P value	Reference
**ebv-miR-BHRF1-2-3p**	0.499225	0.000124	
**hsa-let-7a-3p**	0.109236	5.69E-07	
**hsa-let-7a-5p**	0.168189	2.77E-08	Down[9]
**hsa-let-7c-5p**	0.470644	0.000236	
**hsa-let-7d-3p**	0.302239	0.000256	
**hsa-let-7d-5p**	0.153086	1.5E-09	
**hsa-let-7f-2-3p**	0.232084	2.57E-07	
**hsa-let-7f-5p**	0.058042	6.81E-11	
**hsa-let-7g-5p**	0.219145	5.98E-11	
**hsa-let-7i-5p**	0.424581	3.12E-05	
**hsa-miR-100-5p**	0.48719	0.006004	
**hsa-miR-101-3p**	0.108926	2.53E-09	
**hsa-miR-103a-2-5p**	0.246524	0.000562	
**hsa-miR-103a-3p**	0.093164	4.32E-17	Down[9]
**hsa-miR-106a-5p**	0.012545	1.66E-10	
**hsa-miR-106b-3p**	0.217945	9.75E-08	
**hsa-miR-106b-5p**	0.039878	1.55E-07	Down[9]
**hsa-miR-107**	0.059533	3.06E-12	
**hsa-miR-10a-5p**	0.493143	0.001669	
**hsa-miR-1182**	0.437163	0.005768	
**hsa-miR-125a-5p**	0.330551	1.44E-05	
**hsa-miR-1260b**	0.491123	0.000648	
**hsa-miR-126-3p**	0.060915	9.99E-09	Down[9]
**hsa-miR-126-5p**	0.03083	4.42E-09	
**hsa-miR-1271-5p**	0.133365	0.010988	
**hsa-miR-128-3p**	0.088248	4.62E-10	Down[9]
**hsa-miR-1287-5p**	0.426072	5.46E-05	
**hsa-miR-129-1-3p**	0.455291	4.09E-06	Down[7]
**hsa-miR-1297**	0.073553	2.46E-09	
**hsa-miR-1301-3p**	0.197568	6.34E-05	
**hsa-miR-1304-3p**	0.332879	0.000523	
**hsa-miR-1307-3p**	0.111562	7.63E-06	
**hsa-miR-1307-5p**	0.123755	2.77E-08	
**hsa-miR-130a-3p**	0.014206	1.45E-07	
**hsa-miR-130b-3p**	0.287482	0.000794	Down[9], Down[8]
**hsa-miR-132-3p**	0.221891	2.79E-06	
**hsa-miR-133b**	0.403086	7.71E-05	
**hsa-miR-136-3p**	0.095175	0.003026	
**hsa-miR-139-5p**	0.110333	4.05E-06	

**hsa-miR-1-3p**	0.046803	2.56E-06	
**hsa-miR-140-3p**	0.242067	1.21E-08	
**hsa-miR-142-5p**	0.025706	1.37E-10	
**hsa-miR-143-3p**	0.116278	6.04E-08	Down[9]
**hsa-miR-144-3p**	0.232432	9.43E-05	
**hsa-miR-144-5p**	0.14145	3.77E-06	
**hsa-miR-145-3p**	0.10742	9.59E-06	
**hsa-miR-146a-5p**	0.145801	2.23E-10	Down[9]
**hsa-miR-146b-5p**	0.260523	4.64E-10	
**hsa-miR-148a-3p**	0.041365	3.14E-11	Down[9]
**hsa-miR-148a-5p**	0.214236	1.3E-05	
**hsa-miR-148b-3p**	0.098967	1.27E-10	
**hsa-miR-151a-3p**	0.132894	8.65E-10	
**hsa-miR-151a-5p**	0.135044	2E-15	
**hsa-miR-151a-5p/hsa-miR-151b**	0.436516	4.72E-06	
**hsa-miR-152-3p**	0.090196	2.43E-10	
**hsa-miR-154-5p**	0.4856	0.005079	
**hsa-miR-155-5p**	0.069746	4.06E-09	Down[7]
**hsa-miR-15a-5p**	0.102233	1.18E-06	Down[9]
**hsa-miR-15b-3p**	0.180532	0.000133	
**hsa-miR-15b-5p**	0.038995	1.44E-06	Down[9]
**hsa-miR-16-2-3p**	0.104059	4.73E-08	
**hsa-miR-16-5p**	0.072932	3.32E-07	Down[9]
**hsa-miR-17-3p**	0.18771	0.000682	
**hsa-miR-17-5p**	0.011988	1.12E-09	Down[9]
**hsa-miR-181a-2-3p**	0.253629	0.000547	
**hsa-miR-181a-3p**	0.115612	0.000201	
**hsa-miR-181a-5p**	0.060289	2.23E-12	
**hsa-miR-181b-5p**	0.265639	1.54E-06	
**hsa-miR-181c-3p**	0.01736	1.83E-06	
**hsa-miR-181c-5p**	0.119377	0.002203	
**hsa-miR-181d-5p**	0.312683	0.000256	
**hsa-miR-183-5p**	0.173818	4.4E-06	
**hsa-miR-185-5p**	0.176061	3.48E-06	Down[10]
**hsa-miR-186-5p**	0.042465	7.74E-11	
**hsa-miR-191-5p**	0.193247	4.04E-12	Down[9]
**hsa-miR-192-5p**	0.251385	1.11E-06	Down[9]
**hsa-miR-195-5p**	0.232902	0.000279	Down[9]
**hsa-miR-197-3p**	0.372574	8.68E-05	
**hsa-miR-198**	0.361836	0.001181	
**hsa-miR-199a-3p/hsa-miR-199b-3p**	0.054529	3.52E-11	Down[10]
**hsa-miR-199a-5p**	0.019187	4.21E-08	
**hsa-miR-19a-3p**	0.11396	4.27E-08	
**hsa-miR-19b-3p**	0.075732	3.69E-08	Down[9], Down[11]

**hsa-miR-200c-3p**	0.2686	6.45E-07	
**hsa-miR-203a-3p**	0.217502	2.66E-06	
**hsa-miR-208b-3p**	0.411088	0.014372	
**hsa-miR-20a-5p**	0.01492	1.53E-07	Down[9]
**hsa-miR-20b-5p**	0.026574	2.04E-08	
**hsa-miR-210-3p**	0.262643	0.000238	
**hsa-miR-21-3p**	0.036548	2.01E-07	
**hsa-miR-21-5p**	0.145234	2.12E-13	Down[9], UP[7]
**hsa-miR-219a-5p**	0.3054	1.77E-06	
**hsa-miR-221-3p**	0.434022	8.23E-08	Down[9], Down[8]
**hsa-miR-222-3p**	0.16941	6.24E-05	Down[9]
**hsa-miR-223-3p**	0.19626	2.23E-12	Down[9]
**hsa-miR-223-5p**	0.15403	2.17E-05	
**hsa-miR-22-3p**	0.378569	7.26E-05	Down[9]
**hsa-miR-224-5p**	0.041217	2.14E-07	
**hsa-miR-22-5p**	0.158347	6.31E-08	
**hsa-miR-23a-3p**	0.112153	3.38E-14	Down[9], Down[8]
**hsa-miR-23b-3p**	0.148884	3.17E-12	
**hsa-miR-23b-5p**	0.473325	0.036469	
**hsa-miR-23c**	0.453318	8.79E-06	
**hsa-miR-24-3p**	0.127439	1.35E-09	Down[9]
**hsa-miR-25-3p**	0.291899	9.68E-06	Down[9]
**hsa-miR-2682-5p**	0.437084	0.041644	
**hsa-miR-26a-1-3p**	0.19005	2.64E-05	
**hsa-miR-26a-2-3p**	0.290187	9.02E-08	
**hsa-miR-26a-5p**	0.014954	5.74E-12	Down[9]
**hsa-miR-26b-3p**	0.179949	7.97E-05	
**hsa-miR-26b-5p**	0.043202	1.98E-09	Down[9]
**hsa-miR-27a-3p**	0.048543	2.04E-08	Down[9]
**hsa-miR-27b-3p**	0.022404	1.31E-10	Down[10]
**hsa-miR-27b-5p**	0.325828	0.000814	
**hsa-miR-28-3p**	0.115984	8.3E-08	
**hsa-miR-28-5p**	0.085721	5.28E-09	
**hsa-miR-29c-5p**	0.117644	1.23E-05	
**hsa-miR-30a-5p**	0.084782	1.11E-14	
**hsa-miR-30b-5p**	0.016978	7.6E-11	Down[10]
**hsa-miR-30c-5p**	0.158201	1.29E-06	
**hsa-miR-30d-5p**	0.128303	3.57E-16	Down[9], Down[8]
**hsa-miR-30e-3p**	0.079369	1.12E-08	
**hsa-miR-30e-5p**	0.106516	9.22E-13	Down[9]
**hsa-miR-3120-3p**	0.092914	0.002139	
**hsa-miR-3133**	0.211391	7.92E-08	
**hsa-miR-3141**	0.467857	0.040515	
**hsa-miR-3156-5p**	0.378923	0.0062	

**hsa-miR-3174**	0.399	1.03E-07	
**hsa-miR-3177-3p**	0.065486	1.73E-06	
**hsa-miR-3198**	0.488704	0.01414	
**hsa-miR-323a-3p**	0.329899	1.86E-06	
**hsa-miR-323b-3p**	0.224971	0.00013	
**hsa-miR-326**	0.280704	0.00046	
**hsa-miR-328-5p**	0.454063	0.000534	
**hsa-miR-329-3p**	0.079423	0.008081	
**hsa-miR-330-3p**	0.067336	0.00012	
**hsa-miR-331-3p**	0.070589	3.3E-08	
**hsa-miR-335-5p**	0.137252	1.33E-09	
**hsa-miR-338-5p**	0.121408	4.09E-08	
**hsa-miR-339-3p**	0.181539	6.45E-07	
**hsa-miR-339-5p**	0.056182	1.32E-07	
**hsa-miR-340-5p**	0.096734	1.14E-06	
**hsa-miR-342-3p**	0.302213	0.000665	Down[10]
**hsa-miR-3529-3p**	0.116132	5.45E-07	
**hsa-miR-3610**	0.461693	0.023507	
**hsa-miR-361-5p**	0.087857	2.47E-10	
**hsa-miR-3622a-5p**	0.252046	0.045521	
**hsa-miR-363-3p**	0.09303	1.48E-07	
**hsa-miR-3678-3p**	0.367595	0.015926	
**hsa-miR-3679-5p**	0.473954	0.001531	
**hsa-miR-369-3p**	0.061221	3.4E-06	
**hsa-miR-370-3p**	0.283595	0.001871	
**hsa-miR-374a-3p**	0.165973	0.000137	
**hsa-miR-374a-5p**	0.140951	6.95E-07	Down[9]
**hsa-miR-374b-5p**	0.145522	1.69E-06	
**hsa-miR-374c-3p**	0.475381	6.26E-10	
**hsa-miR-374c-5p**	0.123869	0.002289	
**hsa-miR-378c**	0.260069	5.41E-05	
**hsa-miR-378e**	0.263943	1.35E-06	
**hsa-miR-379-5p**	0.071113	6.22E-07	
**hsa-miR-382-3p**	0.164676	1.97E-07	
**hsa-miR-3928-3p**	0.068795	5.23E-06	
**hsa-miR-409-3p**	0.24217	1.91E-05	
**hsa-miR-410-3p**	0.108797	6.73E-05	
**hsa-miR-411-3p**	0.360319	0.004429	
**hsa-miR-411-5p**	0.128003	0.000242	
**hsa-miR-421**	0.119241	8E-11	
**hsa-miR-423-3p**	0.411044	4.27E-05	
**hsa-miR-424-3p**	0.19856	1.18E-07	
**hsa-miR-425-3p**	0.158692	2.02E-09	
**hsa-miR-425-5p**	0.148898	2.11E-10	

**hsa-miR-4288**	0.316926	4.46E-12
**hsa-miR-4289**	0.065962	2.37E-10
**hsa-miR-4291**	0.177469	2.42E-07
**hsa-miR-4306**	0.238701	1.16E-09
**hsa-miR-4317**	0.189915	2.41E-10
**hsa-miR-4318**	0.099105	1.34E-05
**hsa-miR-4325**	0.103961	3.12E-09
**hsa-miR-4328**	0.389842	0.004705
**hsa-miR-4423-3p**	0.376302	0.004491
**hsa-miR-4433a-3p**	0.267874	0.00046
**hsa-miR-4436b-3p**	0.319482	0.007618
**hsa-miR-4446-3p**	0.129816	1.47E-06
**hsa-miR-4461**	0.308076	8.8E-05
**hsa-miR-4500**	0.151936	1.35E-10
**hsa-miR-4516**	0.465827	0.015251
**hsa-miR-451a**	0.240762	4.18E-08
**hsa-miR-452-5p**	0.433541	0.003063
**hsa-miR-4641**	0.494259	0.021572
**hsa-miR-4655-5p**	0.22944	0.005888
**hsa-miR-4659b-3p**	0.193892	7.7E-07
**hsa-miR-4662a-5p**	0.135997	1.39E-06
**hsa-miR-4676-3p**	0.18677	0.00394
**hsa-miR-4692**	0.116783	4.17E-09
**hsa-miR-4708-5p**	0.364118	0.028607
**hsa-miR-4714-3p**	0.387269	0.008414
**hsa-miR-4769-5p**	0.437531	0.004953
**hsa-miR-4773**	0.425773	0.045809
**hsa-miR-485-5p**	0.373702	0.00031
**hsa-miR-487a-3p**	0.35981	6.54E-07
**hsa-miR-487b-3p**	0.07645	5.35E-06
**hsa-miR-493-3p**	0.448689	0.020796
**hsa-miR-493-5p**	0.019623	3.17E-07
**hsa-miR-494-3p**	0.093717	0.000123
**hsa-miR-495-3p**	0.065546	0.000103
**hsa-miR-5007-3p**	0.269255	0.000184
**hsa-miR-500a-3p**	0.170816	0.001117
**hsa-miR-5010-5p**	0.176183	0.000743
**hsa-miR-501-3p**	0.4867	0.003387
**hsa-miR-502-3p**	0.14555	1.31E-05
**hsa-miR-505-3p**	0.110447	1.83E-06
**hsa-miR-5089-5p**	0.400849	0.004558
**hsa-miR-532-5p**	0.052714	1.63E-07
**hsa-miR-539-3p**	0.156758	0.000453
**hsa-miR-542-3p**	0.158451	0.000531

**hsa-miR-543**	0.123421	0.000131	
**hsa-miR-548d-5p**	0.212852	4.94E-05	
**hsa-miR-548e-3p**	0.155152	2.49E-08	
**hsa-miR-548l**	0.131854	0.00427	
**hsa-miR-548v**	0.250801	6.5E-07	
**hsa-miR-556-3p**	0.340135	6.86E-05	
**hsa-miR-5579-3p**	0.392462	0.002202	
**hsa-miR-584-5p**	0.162214	5.64E-06	
**hsa-miR-598-3p**	0.467893	0.005103	
**hsa-miR-599**	0.25624	8.95E-07	
**hsa-miR-628-3p**	0.179411	3.86E-08	
**hsa-miR-641**	0.325968	1.72E-05	
**hsa-miR-652-3p**	0.04434	1.69E-07	
**hsa-miR-654-3p**	0.117243	3.3E-05	
**hsa-miR-654-5p**	0.291947	2.65E-05	
**hsa-miR-656-3p**	0.434022	5.45E-05	
**hsa-miR-660-5p**	0.182212	3.42E-08	
**hsa-miR-664a-5p**	0.173678	0.000278	
**hsa-miR-671-3p**	0.160911	0.000135	
**hsa-miR-708-3p**	0.169687	6.52E-10	
**hsa-miR-708-5p**	0.34214	0.000139	
**hsa-miR-7-1-3p**	0.078685	7.74E-09	
**hsa-miR-744-5p**	0.164206	8.8E-08	UP[7]
**hsa-miR-7-5p**	0.370035	0.000538	
**hsa-miR-769-5p**	0.162319	0.000468	
**hsa-miR-889-3p**	0.080718	5.7E-06	
**hsa-miR-92a-3p**	0.182562	4.58E-12	Down[9]
**hsa-miR-92b-3p**	0.115382	5.36E-12	
**hsa-miR-93-3p**	0.20325	0.001128	
**hsa-miR-93-5p**	0.101397	2.18E-10	
**hsa-miR-96-5p**	0.166978	1.07E-06	
**hsa-miR-98-3p**	0.128474	3.32E-08	
**hsa-miR-98-5p**	0.101131	6.66E-10	
**hsa-miR-99b-5p**	0.218216	0.000266	
**hsa-miRPlus-A1086**	0.348481	0.008195	
**hsv1-miR-H5-3p**	0.080535	3.66E-12	
**hsv2-miR-H9-5p**	0.439575	0.028362	

UP, upregulated; Down, down-regulated; ND, no difference

Supplemental Table 8. Characteristics of the validation cohort.

	Controls(n=45)	Chronic heart failure(n=45)	P value
Age (years)	57.7±9.2	60.8±12.2	0.167
Male/female (n/n)	24/21	25/20	0.832
LVEF (%)	67.5±6.2	41.6±13.5***	<0.0001
LVEDD (mm)	42.1±10.9	58.1±11.5***	<0.0001
NT-proBNP (ng/L)	82.3±77.2	5245± 5678***	<0.0001
SBP (mmHg)	129±14	131±25	0.67
DBP(mmHg)	79±10	80±15	0.69
Glucose (mmol/L)	6.1±2.4	6.7±2.5	0.21
TG (mmol/L)	1.4±0.8	1.2±0.6	0.16
TC (mmol/L)	4.1±0.7	3.9±0.9	0.41
HDL (mmol/L)	1.07±0.27	0.94±0.26*	0.02
LDL (mmol/L)	2.46±0.71	2.37±0.89	0.60

LVEF, left-ventricular ejection fraction; LVEDD, left ventricular end diastolic diameter; SBP, systolic blood pressure; DPB, diastolic blood pressure; TG, total **triglyceride;** TC, total cholesterol; HDL, high-density lipoprotein; LDL, low-density lipoprotein; *p<0.05, *** p<0.0001

Supplemental Table 9. Profile data of the reported cardiomyocytes-derived miRNAs.

miRNA	Fold change (HF vs Control) (Heart)	Fold change (HF vs Control) (Plasma)	P value
**hsa-miR-499**	**No difference**	**No difference**	
hsa-miR-1	**No difference**	0.047	2.56E-06
hsa-miR-208	**No difference**	0.411	0.014
hsa-miR-133	**No difference**	0.403	7.71E-05
hsa-miR-30c	**No difference**	0.158	1.29E-06
has-miR-378	**No difference**	0.260	5.41E-05
hsa-miR-181c	**No difference**	0.017	1.83E-06

Supplemental Table 10. AUC of 10 successfully validated miRNAs.

miRNA	AUC (95% CI)
**miR-660-3p**	0.984(0.966-1.002)
**miR-665**	0.979(0.956-1.003)
**miR-1285-3p**	0.951(0.911-0.990)
**miR-4491**	0.978(0.950-1.007)
**miR-206**	0.947(0.898-0.995)
**miR-1268b**	0.994(0.982-1.005)
**miR-130a-3p**	0.659(0.547-0.771)
**miR-330-3p**	0.611(0.490-0.733)
**miR-30c-5p**	0.724(0.617-0.831)
**miR-181c-3p**	0.771(0.673-0.869)

AUC, area under the receiver operating characteristic curve

Supplemental Table 11. Summarized data of all selected miRNAs

miRNA	Profiles (heart) HF vs. Con	Profiles (plasma) HF vs. Con	QRT-PCR (plasma) HF vs. Con	cardiac enriched	CF/CM enriched	AUC	Correlation to EF%
**miR-660-3p**	**UP**	**UP**	**UP**	**Yes**	**CF**	**0.984**	**Yes**
**miR-665**	**UP**	**UP**	**UP**	**Yes**	**CF**	**0.979**	**Yes**
**miR-1285-3p**	**UP**	**UP**	**UP**	**Yes**	**CF**	**0.951**	**Yes**
**miR-4491**	**UP**	**UP**	**UP**	**Yes**	**CF**	**0.978**	No
miR-206	Down	UP	UP	No	CF	0.947	No
miR-1268b	Down	UP	UP	No	CF	0.994	No
**miR-130a-3p**	UP	Down	Down	Yes	CM	0.659	No
**miR-330-3p**	UP	Down	Down	No	CF	0.611	No
**miR-30c-5p**	ND	Down	Down	Yes	CM	0.724	No
**miR-181c**	ND	Down	Down	Yes	CM	0.771	No
miR-199a-5p	UP	Down	ND	No			No
miR-221-3p	Down	Down	ND	Yes			No
miR-487b-3p	Down	Down	ND	No			No
miR-4288	Down	Down	ND	No			No

UP, upregulated; Down, down-regulated; ND, no difference; CF, cardiac fibroblasts; CM, cardiomyocytes; AUC, area under the receiver operating characteristic curve

References

1. Yan M, Chen C, Gong W, Yin Z, Zhou L, Chaugai S, Wang DW. miR-21-3p regulates cardiac hypertrophic response by targeting histone deacetylase-8. Cardiovasc Res 2015;**105**(3):340-52.
2. Ikeda S, Kong SW, Lu J, Bisping E, Zhang H, Allen PD, Golub TR, Pieske B, Pu WT. Altered microRNA expression in human heart disease. Physiol Genomics 2007;**31**(3):367-73.
3. van Rooij E, Sutherland LB, Liu N, Williams AH, McAnally J, Gerard RD, Richardson JA, Olson EN. A signature pattern of stress-responsive microRNAs that can evoke cardiac hypertrophy and heart failure. Proc Natl Acad Sci U S A 2006;**103**(48):18255-60.
4. Matkovich SJ, Van Booven DJ, Youker KA, Torre-Amione G, Diwan A, Eschenbacher WH, Dorn LE, Watson MA, Margulies KB, Dorn GW, 2nd. Reciprocal regulation of myocardial microRNAs and messenger RNA in human cardiomyopathy and reversal of the microRNA signature by biomechanical support. Circulation 2009;**119**(9):1263-71.
5. Akat KM, Moore-McGriff D, Morozov P, Brown M, Gogakos T, Correa Da Rosa J, Mihailovic A, Sauer M, Ji R, Ramarathnam A, Totary-Jain H, Williams Z, Tuschl T, Schulze PC. Comparative RNA-sequencing analysis of myocardial and circulating small RNAs in human heart failure and their utility as biomarkers. Proc Natl Acad Sci U S A 2014;**111**(30):11151-6.
6. Eskildsen TV, Schneider M, Sandberg MB, Skov V, Bronnum H, Thomassen M, Kruse TA, Andersen DC, Sheikh SP. The microRNA-132/212 family fine-tunes multiple targets in Angiotensin II signalling in cardiac fibroblasts. J Renin Angiotensin Aldosterone Syst 2014.
7. Cakmak HA, Coskunpinar E, Ikitimur B, Barman HA, Karadag B, Tiryakioglu NO, Kahraman K, Vural VA. The prognostic value of circulating microRNAs in heart failure: preliminary results from a genome-wide expression study. J Cardiovasc Med (Hagerstown) 2015;**16**(6):431-7.
8. Watson CJ, Gupta SK, O'Connell E, Thum S, Glezeva N, Fendrich J, Gallagher J, Ledwidge M, Grote-Levi L, McDonald K, Thum T. MicroRNA signatures differentiate preserved from reduced ejection fraction heart failure. Eur J Heart Fail 2015;**17**(4):405-15.
9. Marfella R, Di Filippo C, Potenza N, Sardu C, Rizzo MR, Siniscalchi M, Musacchio E, Barbieri M, Mauro C, Mosca N, Solimene F, Mottola MT, Russo A, Rossi F, Paolisso G, D'Amico M. Circulating microRNA changes in heart failure patients treated with cardiac resynchronization therapy: responders vs. non-responders. Eur J Heart Fail 2013;**15**(11):1277-88.
10. Ellis KL, Cameron VA, Troughton RW, Frampton CM, Ellmers LJ, Richards AM. Circulating microRNAs as candidate markers to distinguish heart failure in breathless patients. Eur J Heart Fail 2013;**15**(10):1138-47.

11. van Almen GC, Verhesen W, van Leeuwen RE, van de Vrie M, Eurlings C, Schellings MW, Swinnen M, Cleutjens JP, van Zandvoort MA, Heymans S, Schroen B. MicroRNA-18 and microRNA-19 regulate CTGF and TSP-1 expression in age-related heart failure. Aging Cell 2011;**10**(5):769-79.
